# Single Cell Kinetics of Phenotypic Switching in the Arabinose Utilization System of *E. coli*

**DOI:** 10.1371/journal.pone.0089532

**Published:** 2014-02-26

**Authors:** Georg Fritz, Judith A. Megerle, Sonja A. Westermayer, Delia Brick, Ralf Heermann, Kirsten Jung, Joachim O. Rädler, Ulrich Gerland

**Affiliations:** 1 Arnold Sommerfeld Center for Theoretical Physics and CeNS, Ludwig- Maximilians-Universität München, Munich, Germany; 2 Center for Integrated Protein Science (CiPSM) at the Department of Biology, Microbiology, Ludwig-Maximilians-Universität München, Martinsried, Germany; 3 Faculty of Physics and CeNS, Ludwig-Maximilians-Universität München, Munich, Germany; Baylor College of Medicine, United States of America

## Abstract

Inducible switching between phenotypes is a common strategy of bacteria to adapt to fluctuating environments. Here, we analyze the switching kinetics of a paradigmatic inducible system, the arabinose utilization system in *E. coli.* Using time-lapse fluorescence microscopy of microcolonies in a microfluidic chamber, which permits sudden up- and down-shifts in the inducer arabinose, we characterize the single-cell gene expression dynamics of the *araBAD* operon responsible for arabinose degradation. While there is significant, inducer-dependent cell-to-cell variation in the timing of the on-switching, the off-switching triggered by sudden removal of arabinose is homogeneous and rapid. We find that rapid off-switching does not depend on internal arabinose degradation. Because the system is regulated via the internal arabinose level sensed by AraC, internal arabinose must be rapidly depleted by leakage or export from the cell, or by degradation via a non-canonical pathway. We explored whether the poorly characterized membrane protein AraJ, which is part of the arabinose regulon and has been annotated as a possible arabinose efflux protein, is responsible for rapid depletion. However, we find that AraJ is not essential for rapid switching to the off-state. We develop a mathematical model for the arabinose system, which quantitatively describes both the heterogeneous on-switching and the homogeneous off-switching. The model also predicts that mutations which disrupt the positive feedback of internal arabinose on the production of arabinose uptake proteins change the heterogeneous on-switching behavior into a homogeneous, graded response. We construct such a mutant and confirm the graded response experimentally. Taken together, our results indicate that the physiological switching behavior of this sugar utilization system is asymmetric, such that off-switching is always rapid and homogeneous, while on-switching is slow and heterogeneously timed at sub-saturating inducer levels.

## Introduction

Signaling pathways and gene regulatory circuits enable bacteria to respond to environmental changes by turning functional genetic modules on or off. Examples for such modules include inducible carbon utilization systems [1], [2], cell-to-cell communication systems [3]–[5], and stress response systems [6]–[8]. In many cases, noise in gene expression strongly influences the switching process [9], [10]. Owing to recent technological advances, the gene expression dynamics of functional modules in single cells of bacterial populations can now be monitored in real time [11] while their environments can be controlled using microfluidic devices [12], [13]. The resulting experimental portrait of these systems, combined with theoretical analysis, can yield unprecedented insight into the strategies for cellular decision making [14].

The inducible sugar utilization systems of *E. coli* display a clearcut switching behavior and are well suited for quantitative analysis. The lactose (*lac*) and the arabinose (*ara*) system in particular are paradigmatic examples of negative and positive transcription regulation, respectively, and are well characterized at the molecular level [1], [2]. In both systems, an “all-or-nothing” switch-like gene expression behavior has been observed and analyzed on the single-cell level [15]–[23]. For the arabinose system, we previously characterized the single-cell induction dynamics using time-lapse fluorescence microscopy and identified a heterogeneous timing behavior, where the time point of gene induction is variable within a clonal population [23]. This heterogeneity was found to depend sensitively on the external arabinose concentration, with highly variable timing at low arabinose concentrations and nearly simultaneous switching at high concentrations. Theoretical analysis of the underlying regulatory circuit revealed that cell-to-cell variability in the number of arabinose transporters at the time of sugar addition could consistently account for the observations. However, the single-cell induction kinetics has so far only been investigated in strains unable to metabolize arabinose [23], i.e., it is unclear whether heterogeneous timing is a physiological behavior of *E. coli.*

**Figure 1:**
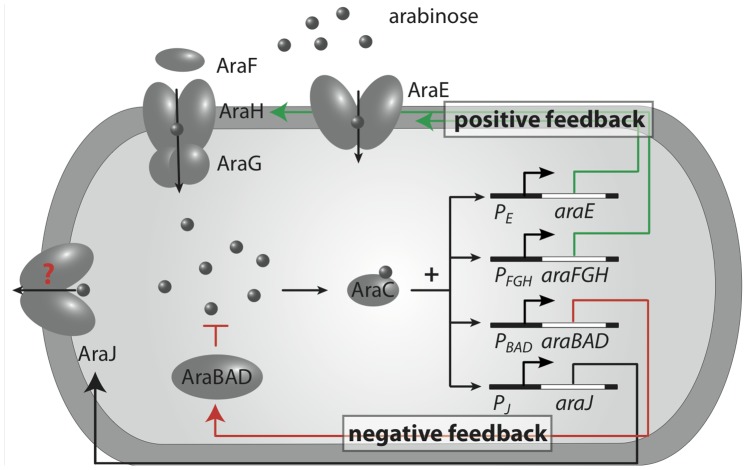
Scheme of the arabinose utilization system in *E. coli*. Arabinose is imported via the arabinose transporters AraE and AraFGH. If the intracellular arabinose level is sufficiently large, arabinose binds the transcriptional regulator AraC. This complex activates the promoters P*_E_*, P*_FGH_*, P*_BAD_* and P*_J_*, driving expression of *araE*, *araFGH*, *araBAD* and *araJ*, respectively. The latter two operons encode proteins for arabinose catabolism (AraBAD) and a putative arabinose efflux pump (AraJ). The negative autoregulation of AraC resulting in homeostatic control of its own level is not shown explicitly. Arrows indicate arabinose transport and positive regulation, whereas the T-shaped arrow indicates arabinose metabolization.

Here, we analyze the single-cell switching of different *E. coli* strains with and without the native arabinose degradation capability. We first test whether heterogeneous timing in the on-switching is indeed a robust phenomenon. We then characterize the switching kinetics of induced cells back into the off state after sudden removal of arabinose. These kinetics are intimately coupled to the depletion of intracellular arabinose, since the activation of the *ara* system depends on the internal arabinose level sensed by the regulator AraC (Fig. 1). We investigate whether a poorly characterized arabinose-inducible gene, *araJ*, which displays sequence similarity with drug efflux proteins [24] and could potentially function as a sugar efflux system [25], plays a significant role in the depletion process. We analyze our data with a refined mathematical model for the dynamics of the *ara* system, which incorporates the arabinose depletion process. The model predicts that deletion of the positive feedback loop of arabinose uptake proteins on their own synthesis (see Fig. 1) would completely alter the induction behavior of the *ara* system, turning the binary, switch-like response to a graded response. We confirm this prediction experimentally using a mutant strain with an arabinose-independent basal expression of *araE* encoding the arabinose transporter AraE. Taken together, this study provides a detailed quantitative characterization of the single-cell switching dynamics of the arabinose system.

## Materials and Methods

### Bacterial Strains and Plasmid

In this study *E.coli* strains MG1655 (wild-type), BW25113 (Δ*araBAD*), JW0386-1 (Δ*araBAD* Δ*araJ*), JW1889-3 (Δ*araBAD* Δ*araFGH*) and JW1889-5 (Δ*araBAD* Δ*araFGH* P*_araE_*::P*_lac_*) were used (see Table S1 and Text S1 for details). All strains were transformed with the reporter plasmid pBAD24-GFP [23], containing the gene encoding *gfpmut3* [26] under control of the P*_BAD_* promoter as well as the *araC* gene.

### Growth Conditions and Induction Experiments

Overnight pre-cultures were inoculated from single colonies grown on LB [27] agar plates (1.5% (w/w)). LB agar plates and overnight pre-cultures contained ampicillin. Cultures were inoculated 1∶400 from precultures, subsequently grown for 3 to 4h (OD600 ∼ 0.1–0.3) and then prepared for microscopy. All liquid cultures were grown at 37°C in M63 medium [28] containing 0.2% (v/v) (JW1889-3, JW1889-5) or 0.5% (v/v) (all other experiments) glycerol as C-source. For microscopy, bacteria were applied to one channel of a poly-L-lysine coated microfluidic chamber (µ-slide VI; Ibidi, Martinsried, Germany). The slide was subsequently incubated at 37°C for several minutes, rinsed with fresh, pre-warmed medium and transferred to the microscope. For induction experiments of strains JW1889-3 and JW1889-5 the channel was flushed several times with pre-warmed medium containing the desired arabinose concentration. A slightly different procedure was used for the strains MG1655, BW25113 and JW0386-1 in order to obtain comparable results: In experiments with arabinose-degrading strains arabinose also needs to be supplied during the experiment after the initial addition in order to keep the concentration constant. This was either achieved by regular manual rinsing or by using a custom built flow system. To generate arabinose pulses, arabinose was removed at the indicated time after induction by rinsing the channel with pre-warmed medium without arabinose, which was subsequently supplied until the end of the experiment.

### Time-lapse Microscopy and Image Analysis

Time-lapse microscopy was performed as previously described [23]. Briefly, bright-field and fluorescence images of several fields in one sample were acquired every 5 min at 100×magnification. Image analysis was performed in a semi-automated way using the ImageJ PlugIn CellEvaluator [29]. Using this program, bacterial outlines were determined in the bright-field images and tracked through the entire time-series. The outlines were then transferred to the background-corrected fluorescence images in order to extract the time-courses of the area (number of pixels), representing bacterial growth, and of the mean fluorescence (sum over all pixel values within the outline divided by the number of pixels), representing gene expression.

### Mathematical Model for Switching Dynamics in the Arabinose System

To extract empirical delay times for gene expression in individual cells, we used the same heuristic mathematical model for the fluorescence trajectories as was previously used in [23]. This model is based on the assumption of a step-like increase of the *gfp* transcription rate from its basal to its maximal value at a certain time point. By fitting the model to the experimental fluorescence trajectories, this time point is estimated separately for each cell and the delay time determined as the time difference to the arabinose upshift.

For all other analyses, we used the more detailed set of rate equations described in the following to model the single-cell dynamics of internal arabinose and gene regulation. Within this model, the internal arabinose concentration, a(t), is described by
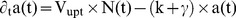
(1a)where N(t) denotes the concentration of arabinose transporters and V_upt_ the uptake rate per transporter. Comparison of arabinose uptake in wild-type strains with *araE* and *araFGH* deletion strains revealed that these two arabinose transporters do not operate independently and that AraE is the dominant uptake protein for intermediate to high sugar levels [30]. Therefore our model considers only AraE as uptake protein for arabinose import. The rate of sugar uptake, V_upt_, depends on the external sugar concentration, a_ex_, and the Michealis-Menten constant, K_m_, and maximal transport rate, V_max_, of AraE,
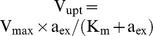
. The second term on the right hand side of Eq. 1 accounts for all processes that reduce the internal arabinose concentration. As we monitor cell growth on the single-cell level, the cell growth rate γ enters explicitly (growth dilutes the internal arabinose), whereas the rate constant *k* subsumes all other loss processes: arabinose degradation (in strains with intact *araBAD* operon), arabinose leakage via AraE or other transporters [31], and possibly the action of active sugar export systems or non-canonical metabolic pathways. In Eq. (1), we have assumed that the loss of internal arabinose follows first-order reaction kinetics, consistent with the data of Novotny and Englesberg [31]. Eq. (1) also assumes that the loss rate is constant, independent of the concentration of transporters, N(t). We also consider an alternative version of the model where the rate *k* is taken to be proportional to N(t),



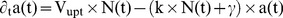
(1b)These two alternatives should be regarded as the extremes of a continuum of models where the loss is partly dependent on N(t) and partly independent. We will see, however, that our data does not allow us to discriminate between these cases.

The gene regulation of the arabinose system is mediated by the transcription factor AraC. In the presence of sufficient amounts of internal arabinose, AraC stimulates transcription from the promoters P*_E_* and P*_BAD_*. The promoter activity of P*_BAD_* was found to increase cubically with the internal arabinose concentration [32]. While the quantitative characteristics of P*_E_* are not known so far, the high similarity between P*_E_* and P*_BAD_* suggests that the input-output relations of both promoters are comparable [33]. Hence, we model transcriptional regulation in both cases as Hill functions with Hill coefficient three,
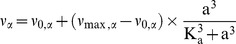
(2)where ν_0,α_ is the basal and ν_max,α_ the maximal transcription rate of P*_E_* (α = e; driving *araE* expression) and P*_BAD_* (α = g; driving *gfp* expression). We do not consider the dynamics of AraC explicitly, since AraC negatively regulates its own expression [2], resulting in homeostatic control of its concentration. With this, the dynamics of the AraE mRNA concentration, n(t), and the GFP mRNA concentration, g(t), follows



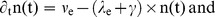
(3)

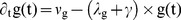
(4)where λ_e_ and λ_g_ are the degradation rates of AraE and GFP mRNA, respectively. Likewise, protein dynamics of the AraE concentration, N(t), the immature GFP concentration, I(t), and the fluorescent GFP concentration, G(t), obey the rate equations



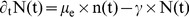
(5)

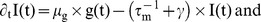
(6)

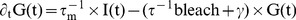
(7)


Here µ_e_ and µ_g_ are the translation rates of AraE and GFP, respectively, τ_m_ is the time constant of GFP maturation, the process whereby the folded GFP becomes fluorescent and τ_bleach_ is the time constant of GFP bleaching. Finally, for a direct comparison of model and experiment we need to consider 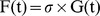 which converts protein concentrations into arbitrary fluorescence units via a scaling factor σ.

### Parameter Estimation

The parameters of the model were determined as described in Text S1. Briefly, the growth rate γ was estimated for each individual cell from the time series of the area detected under the microscope (Fig. S1). The parameters that were expected to display little cell-to-cell variability were fixed to values estimated from the literature or independent experiments, see Table S2. The three key parameters, ν_0,e_, ν_max,g_, and *k,* were estimated by solving the reaction system in Eqs. (1a)–(7) numerically and by using a trust-region reflective Newton method (MATLAB, The MathWorks, Inc.) to minimize the total χ^2^ between experiment and model. The resulting fit-parameters are summarized in Table S3. To test whether there exists a physiological parameter regime, in which the alternative model with arabinose export via AraE (using Eq. (1b) instead of Eq. (1a)) is compatible with our data, the parameter space was screened manually for the three main experimental characteristics reported in this study: (i) Heterogeneous timing of gene induction at low inducer concentrations; (ii) Rapid shut-down of transcription after arabinose removal; (iii) Modulation of expression rate (instead of time delay) with external arabinose concentration in a model with constitutive *araE* expression. Here our analysis indicated that there exists a parameter regime in which all of these phenomena are reproduced (see Section “Analysis of the quantitative model and prediction of a mutant behavior” for more details).

## Results

### Heterogeneous Gene Induction in a Strain with Native Arabinose System

A heterogeneous timing behavior in the switching from the off- to the on-state was previously reported for the *ara* system [23]: After the addition of arabinose to the medium, each cell turns on *araBAD* expression in a switch-like manner, but different cells display different time delays. Both the average delay time and its cell-to-cell variability increase with decreasing arabinose concentration. This behavior was observed in a mutant incapable of arabinose degradation [23]. Arabinose degradation should exert a negative feedback on the switching process, since it reduces the internal arabinose level sensed by the transcription factor AraC, see Fig. 1. To find out whether this feedback alters the behavior of the system, we performed a single-cell switching assay with an *araBAD* native strain. To that end, we used *E. coli* MG1655 transformed with reporter plasmid pBAD24-GFP [23] containing the *araC* gene and the P*_BAD_* promoter-controlled rapidly maturing GFP variant *gfpmut3* [26]. The *araC* gene is supplied on the plasmid to guarantee full functionality of the DNA loop required for repression of P*_BAD_* in the absence of arabinose [2] and to provide the proper stoichiometry of transcription factors and P*_BAD_* promoters.

**Figure 2:**
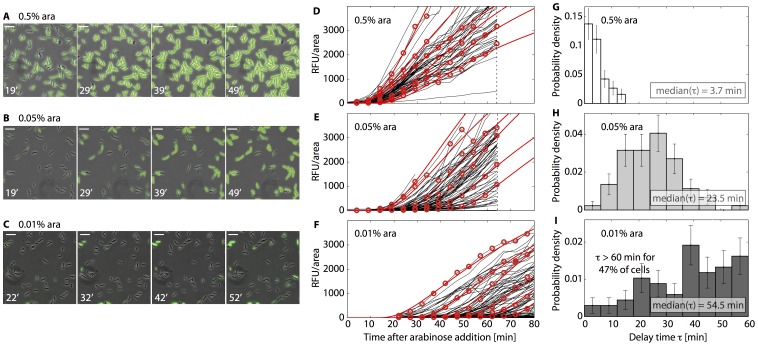
Single-cell switching dynamics from the off- to the on state in the wildtype *ara* system. Bacteria of strain MG1655 were induced with 0.5%, 0.05% or 0.01% arabinose at t = 0 min. The image panels show time series of fluorescence images (A, B, C, scale bar: 5 µm). The single cell fluorescence trajectories (D, E, F) show that fluorescence remains negligible until the so-called delay time at which a strong increase starts. Experimental data points are highlighted by red circles and the corresponding fits are shown as red lines. With decreasing arabinose concentration the delay time increases and varies more significantly between bacteria in a population. This effect is shown quantitatively by the distributions of delay times over the populations (G, H, I), which were extracted by fitting a mathematical function describing the gene expression process. Note that at 0.01% arabinose a significant percentage of the bacteria did not switch on the *ara* system within the experimental observation window. Number of evaluated cells: 63 (0.5% ara), 74 (0.05% ara), 113 (0.01% ara).

Bacteria were attached to the surface of a microfluidic flow chamber and expression of the *ara* system was induced by applying a contiuous flow of minimal medium supplemented with defined concentrations of arabinose. In that, our protocol asserts that extracellular conditions are constant and arabinose is not depleted from the medium. After induction with different arabinose concentrations (0.5%, 0.05% and 0.01%) at t = 0 min, we followed gene expression dynamics by fluorescence time-lapse microscopy (Fig. 2A–C) and determined the mean fluorescence (sum over fluorescence values divided by the number of pixels) in individual cells over time (Fig. 2D–F). For each cell, we then estimated the time delay until promoter activation from the fluorescence time series, using the same method as in [23], see also ‘Materials and Methods’. The resulting distributions of delay times in Fig. 2G-I can therefore be directly compared to the analogous distributions for the Δ*araBAD* mutant in Fig. 5 of ref. [23].

**Figure 5:**
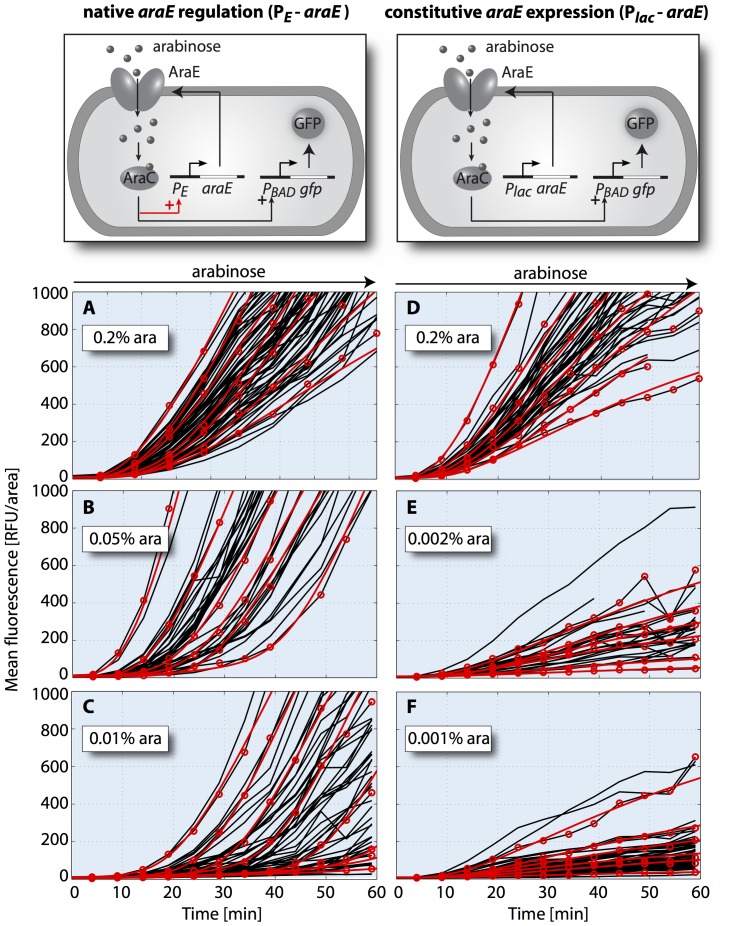
Single-cell induction kinetics of cells with native and constitutive transporter production. Cells of strain JW1889-3 (A–C, native *araE* regulation) and JW1889-5 (D–F, constitutive *araE* expression) containing the reporter plasmid pBAD24-GFP were induced with indicated concentrations of arabinose. Black lines represent the kinetics of the mean fluorescence of individual cells and red circles highlight representative trajectories. Note that some trajectories disappear before the end of the experiment due to detachment of daughter cells after cell division. Red lines are representative fits of our full model of arabinose uptake and gene regulation in Eqs. (1)–(7) to the highlighted experimental trajectories. Number of evaluated cells: 71, 63 and 54 (for 0.2%, 0.05% and 0.01% ara induction of JW1889-3, respectively); 45, 28 and 57 (for 0.2%, 0.002% and 0.001% ara induction of JW1889-5, respectively).

Qualitatively, the observed behavior is very similar to the *araBAD* mutant: At a high arabinose concentration (Fig. 2A,D,G) we find rapid and homogeneous induction within all cells of the population, whereas both the mean and the width of the delay time distribution increase with decreasing arabinose concentration (Fig. 2). The average rates of GFP production are arabinose-independent (Fig. S2), indicating that cells indeed display all-or-none expression behaviour, consistent with previous observations [34]. However, in the wildtype system the median delay times are significantly longer than for the *araBAD* deficient strain at a given (external) arabinose concentration (Table 1). A plausible explanation is that the basal expression of *araBAD* in the native system leads to some arabinose degradation, which further delays the time until the internal arabinose level reaches the threshold for gene activation.

**Table 1:** Median delay times for P*_BAD_* activation. Values for strain LMG194 were taken from ref. [23].

arabinose concentration (w/v)	MG1655 (wild type)	LMG194 (Δ*araBAD*)
0.5%	3.7 min	n/a
0.2%	n/a	4 min
0.05%	23.5 min	7.4 min
0.02%	n/a	16.6 min
0.01%	54.5 min	19.9 min

### Single Cell Expression Kinetics after Arabinose Downshift

**Figure 3:**
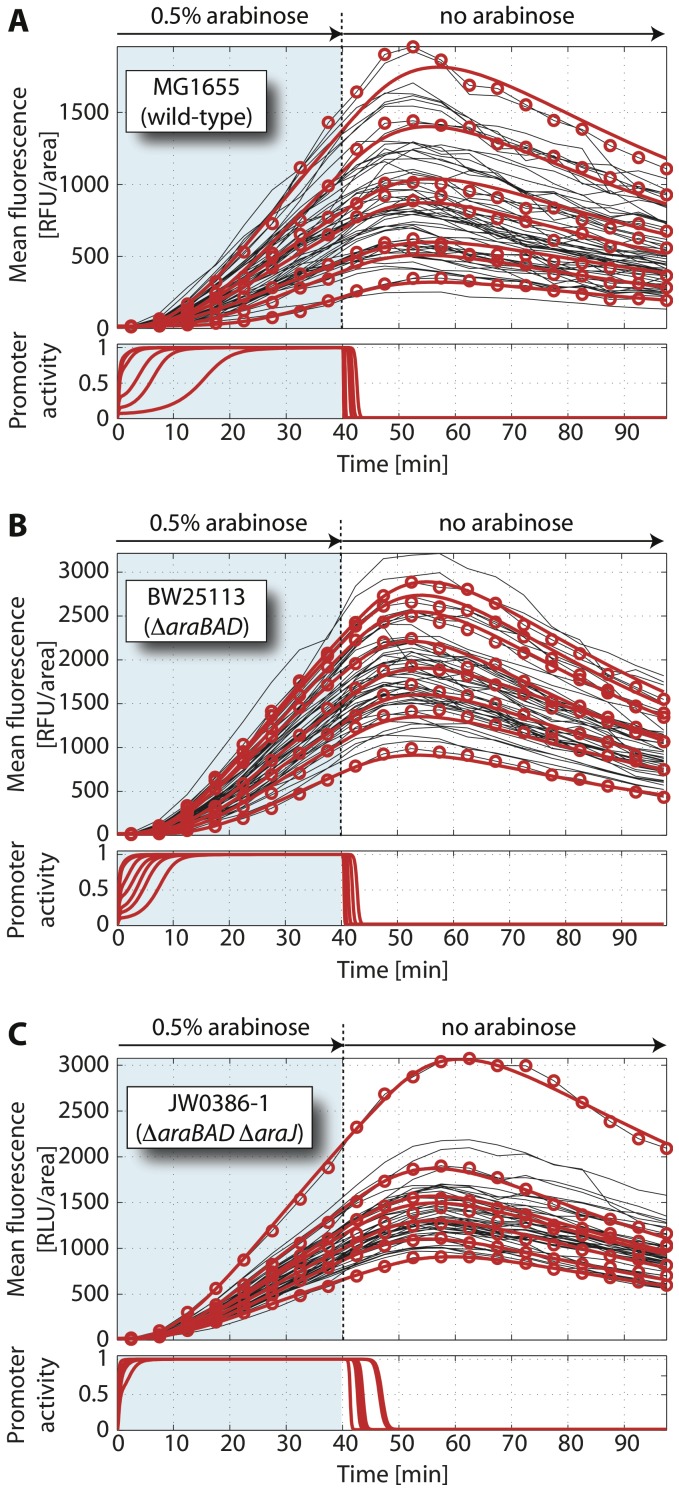
Single-cell response dynamics of the *ara* system upon arabinose down-shift. Initially, cells of strain MG1655 (A, native *ara* regulation), BW25113 (B, Δ*araBAD*) and JW0386-1 (C, Δ*araBAD* and Δ*araJ*) containing the reporter plasmid pBAD24-GFP (*gfpmut3* under the control of the P*_BAD_* promoter) were prepared in the on-state by induction with 0.5% arabinose for 40 min. At this time arabinose was removed by flushing the microfluidic channel with fresh medium without arabinose. (*upper panels*) Black lines are experimental fluorescence trajectories, the red circles highlight the data for representative cells, and the bold red lines correspond to the corresponding fits by the model [Eqs. (1)–(7)] under pulsed addition of arabinose. FU: fluorescence units. (*lower panels*) Red lines show the corresponding dynamics of P*_BAD_* promoter activities in individual cells as inferred from the model. Number of evaluated cells: 62 (strain MG1655), 55 (strain BW25113), 51 (strain JW0386-1). For corresponding dynamics without arabinose removal see Fig. S3.

We next analyzed how the induced wildtype *ara* system responds to a downshift in the external arabinose concentration. To prepare cells in an induced state, the microfluidic channel was rinsed between t = 0 min and t = 40 min with medium containing saturating amounts of arabinose (0.5% arabinose). After 40 min, we removed the external stimulus by flushing with fresh, arabinose-free medium. The resulting single-cell fluorescence trajectories, see Fig. 3A, display an initial increase of mean fluorescence during the induction phase (shaded in blue), reach a maximal fluorescence about 15 min after arabinose removal, and then decrease slowly.

To extract the quantitative characteristics of these single-cell trajectories, we constructed a mathematical model for the single-cell expression dynamics of the *ara* system as described in ‘Materials and Methods’. The smooth shape of the time series in Fig. 3 suggested that the dynamics of individual cells follows a rather deterministic fate, while the differences between the cells stem from cell-to-cell variation of the reaction rates. Therefore, we used a deterministic rate equation model to describe the single-cell dynamics of arabinose uptake, degradation/export and gene regulation, but accounted for cell-to-cell variations in three key model parameters as the major source of cell-to-cell variability in the kinetics of the *ara* system: (i) the number of arabinose importers at the time of sugar addition, determining the time delay until promoter activation, (ii) the production rate of GFP, determining the slope of the fluorescence trajectories, and (iii) an effective first order rate constant, *k*, which accounts for loss of internal arabinose (via degradation or export) and determines the timescale at which cells turn off gene expression after removal of external arabinose. This model refines a previous one in which the induction process of the *ara* system was simply described by a step-like increase of the promoter activity after a certain time delay [23]. Here, we explicitly include the measured dependence of the transcription rates on the internal arabinose concentration [32], which dynamically changes the promoter activity over time. To fit the fluorescence trajectories, we confined the values of the three key model parameters to physiological intervals and fixed the remaining parameters to physiological values (Table S2). See ‘Materials and Methods’ for all details.

Fig. 3A shows model fits (*red lines*) to exemplary fluorescence trajectories (*red circles*). Overall, the model adequately describes the experimental expression kinetics. Interestingly, the model indicates that the time until the fluorescence peaks is largely determined by the maturation time of GFP (<τ_m_> = 6.5 min [23]) and that transcription shuts down after less than 3 min in most cells (Fig. 3A; *lower panel* and Fig. S3). Hence, our data suggests a homogeneous and rapid switching of *ara* gene expression into the off state after arabinose removal. As the level of intracellular arabinose regulates the activity of the P*_BAD_* promoter, a quick shut-off of gene expression should reflect a rapid decrease of the intracellular arabinose level. Our model estimates the associated rate constant *k* to be 2.3 min^−1^ (median value; interquartile range (IQR) from 1.8 to 3.4 min^−1^); see Figs. S4–S6 and Table S3 for an overview of all estimated parameters.

Of course, rapid depletion of internal arabinose is expected in the strain with intact arabinose degradation. We tested whether a strain unable to metabolize arabinose would display a different behavior. We used strain BW25113 with a chromosomal deletion Δ*araBAD*, again harboring the reporter plasmid pBAD24-GFP, and monitored the response of induced bacteria under the same arabinose downshift protocol as above. Similar to the wild-type strain, GFP synthesis ceases quickly after arabinose removal and leads to a maximum of mean fluorescence values about 15 min after arabinose removal, see Fig. 3B. For strain BW25113, the rate constant for arabinose depletion was estimated to be 2.2 min^−1^ (median value; IQR from 1.9 to 3.7 min^−1^) by our model, a value that is only marginally smaller than for the native *ara* system. To verify that switching to the off-state was indeed related to the arabinose down-shift, we exposed the same strain continuously to arabinose and found a steady increase of fluorescence levels throughout the entire observation period (Fig. S3). Hence, our data suggests that arabinose catabolism via AraBAD cannot be the only origin of internal arabinose depletion and concomitant promoter deactivation. Incidentally, our inferred rate constant for arabinose loss is compatible with results from an early biochemical study of the arabinose permease system (*k* = 5.2 min^−1^ [31]). Hence we hypothesized that arabinose loss is mainly due to an export mechanism.

Besides the catabolic genes *araBAD* and the transport systems *araE* and *araFGH,* the arabinose regulon of *E. coli* contains a fourth arabinose-inducible gene [35], *araJ* which appeared interesting for the question at hand, since it displays sequence similarity with drug efflux proteins [24] and it might function as a sugar efflux system [25]. We therefore repeated the arabinose down-shift experiment with a strain (JW0386-1) carrying the pBAD24-GFP plasmid and chromosomal deletions of both *araBAD* and *araJ*. In our microfluidic device, strain JW0386-1 had a slightly increased doubling time compared to MG1655 and BW25113 (Fig. S1 and Table S2). As we monitor and model growth on the single cell level, this change is accounted for in our quantitative analysis. Surprisingly, however, we again observed very similar down-shift behavior as for the native arabinose system. In this case, the estimated rate constant for arabinose loss was 1.4 min^−1^ (median value; IQR from 0.8 to 2.0 min^−1^), only slightly smaller than for the other two strains. Hence, we conclude that AraJ is not the rate-limiting component for internal arabinose depletion either.

Taken together, our results from arabinose down-shift experiments show that the arabinose system is rapidly turned off after removal of external arabinose and that this behavior neither depends on internal arabinose degradation via AraBAD, nor on the activity of the putative membrane protein AraJ. We note, however, that our assay relies on the (comparably) slow maturation of GFP (<τ_m_> = 6.5 min [23]), such that our estimate for the timescale of arabinose loss (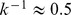 min) merely represents an upper limit and it might well be that the speed of arabinose loss is even faster than this estimate.

### Analysis of the Quantitative Model and Prediction of a Mutant Behavior

**Figure 4:**
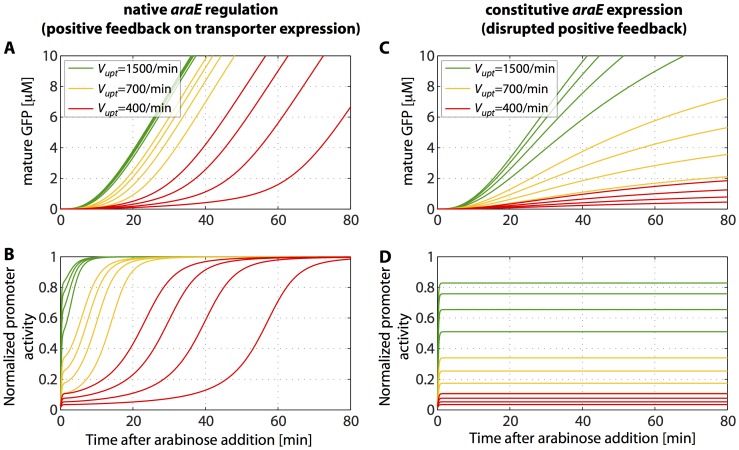
Theoretical gene expression kinetics for native and constitutive transporter production. (A) Fluorescent GFP dynamics and (B) P*_BAD_* promoter activity for native transporter regulation. (C) Fluorescent GFP dynamics and (D) P*_BAD_* promoter activity for constitutive transporter production. Different external arabinose concentrations are represented by various arabinose uptake velocities V_upt_. To illustrate stochastic variability in the initial number of uptake proteins, trajectories are shown for 300, 260, 220 and 180 AraE molecules at each V_upt_. In all cases, the corresponding number of initial AraE molecules of the shown curves decreases from left to right.

Next, we asked whether the same quantitative description used for the downshift experiments of the previous section would also yield a consistent description of the heterogeneous timing phenomenon that we observed for upshifts to sub-saturating arabinose levels. Using the parameter values of Table S2, we calculated the model dynamics for upshifts to three different external arabinose concentrations (leading to different uptake velocities V_upt_ per transporter). To illustrate the effect of the cell-to-cell variation in the number of uptake proteins, we show the model dynamics at each arabinose level for four representative initial uptake protein numbers (300, 260, 220 and 180 proteins), see Fig. 4A. For the highest level of arabinose, all trajectories increase shortly after the upshift, whereas at a low arabinose level the model displays the expected heterogeneous time delay until promoter activation (Fig. 4B). Fig. S7 compares this model behavior with that of the heuristic threshold model in ref. [23], which was previously shown to be quantitatively consistent with the experimental switching kinetics from the off- to the on-state of the *ara* system. The comparison shows that the distribution of delay times has almost the same shape in the two models (Fig. S7C). Also, the scaling of the average delay time with the number of transporters produced in the absence of arabinose displays approximately the same τ_d_ ∼1/N dependence (Fig. S7A), at least over the range of ∼20–200 proteins/cell that we take to represent typical basal levels [20].

So far, we made the model assumption that the loss rate *k* of internal arabinose is independent of the concentration of transporters, N(t), see Eq. (1a). This assumption could be misleading, if a significant fraction of the loss occurs via the arabinose transporters, which are highly expressed in the on-state but only at a low basal level in the off-state. To test to what extent the interpretation of our data depends on this assumption, we also considered a model for the other extreme, where all of the loss occurs via the arabinose transporters, see Eq. (1b). We found that this alternative model is also compatible with all of our data, see Fig. S8. However, only in a narrow parameter regime all phenomena are well reproduced: if the export rate is too low, the rapid transcriptional shut-down after arabinose removal cannot be explained, whereas if the export rate is too high, the model does no longer display heterogeneous timing of gene induction. Hence, we conclude that the incorporation of a depletion mechanism for internal arabinose is essential to explain our data, but we cannot discriminate whether arabinose depletion is constitutive, inducible by arabinose, or a mixture of both (see Discussion).

Note that for the system behavior, the only qualitative difference between the alternative scenarios for arabinose export that we considered concerns the nature of the time delay for induction at sub-saturating external arabinose levels. In either case, full induction of the *araBAD* promoter occurs when the internal arabinose level is sufficiently high to saturate arabinose binding to AraC. However, the time required to reach this level not only depends on the rate of arabinose import, but also on the timescale of transporter synthesis, i.e., the timescale of the positive feedback during the induction process. The scenario with constant rate *k* leads to a clear separation of time scales, with the internal arabinose level following the transporter concentration in a quasi-steady-state. In the other case, export is very slow initially and there is no separation of time scales. However, the simplified interpretation, whereby the delay time is the time for arabinose accumulation to a threshold level inside the cell, is qualitatively correct in both cases.

The positive feedback of Fig. 1 (internal arabinose stimulating increased import of arabinose) is clearly essential for the switch-like behavior of the arabinose system. The precise effect of this feedback can be characterized by comparing with a mutant lacking the positive regulation of the arabinose transporters. Fig. 4C and D show the predicted single-cell induction kinetics of such a mutant, based on the same model as used in Fig. 4A but with constitutive uptake protein production. The system no longer exhibits a clearcut switching to full induction after a well-defined time delay, but instead the promoter activity quickly reaches a constant value according to the internal steady-state value of arabinose, which determines the slope of the fluorescence trajectory. Thus, the model predicts that the fluorescence levels of such a mutant will rise without significant delay but with a sub-maximal slope that depends on the external arabinose level.

### Single-cell Switching in a Mutant with Disrupted Positive Feedback

To experimentally characterize the response of a mutant with disrupted positive feedback, we constructed a strain where *araE* is expressed at a basal level, independent of arabinose, in a Δ*araBAD*Δ*araFGH* background. The construct is based on the *lac* promoter, which is repressed to a constant low level by the *lac* repressor; see Text S1 for details. As a reference strain, we used the same genetic background, but with native, P*_E_*-driven expression of *araE* (Fig. 5; *top panels*). Fluorescence trajectories of the reference strain are shown in Fig. 5A–C for 0.2% (13.3 mM), 0.05% (3.33 mM) and 0.01% (0.66 mM) arabinose. The strain with constitutive *araE* expression was sensitive already at lower arabinose levels, consistent with the fact that the repression of our constructed promoter is relatively leaky. The corresponding fluorescence trajectories are shown for 0.2% (13.3 mM), 0.002% (0.133 mM) and 0.001% (0.066 mM) arabinose (Fig. 5D–F).

A qualitative comparison of the single-cell traces already shows that the dynamics of the two strains differ significantly. In the case of native *araE* regulation (Fig. 5A–C), cells display a distinct lag-phase with marginal fluorescence increase and an induction-phase with high rate of fluorescence production. As the external arabinose concentration decreases, the average as well as the cell-to-cell variation of the delay time increases, qualitatively consistent with the previously described behavior in strain LMG194 [23], a Δ*araBAD* strain in which both *araE* and *araFGH* are functional and under positive feedback control. In contrast, in the strain with constitutive *araE* expression the rate of fluorescence production decreases markedly with decreasing external arabinose, while no significant time delay is visible at any of the tested conditions (Fig. 5D–F). The same picture emerges from a quantitative analysis with the heuristic gene expression model of ref. [23], which assumes a step-like increase of the protein production rate from zero to σ_p_ after a delay time τ_d,_ and determine both parameters by fitting each single-cell trace, see Materials and Methods. The results of this analysis are shown in Table 2. Interestingly, even at saturating arabinose levels (0.2% arabinose) the average protein production rate in the strain without positive feedback is only 60% of the production rate in the reference strain. This observation is consistent with the dynamics in the model without positive feedback (Fig. 4D), for which the promoter activity remains sub-maximal even for very high arabinose uptake velocities.

**Table 2:** Mean and standard deviation of delay time τ_d_ and protein expression rate α_p_ distribution.

relevant genotype	arabinoseconcentration (w/v)	<τ_d_> [min]	σ(τ_d_) [min]	<α_p_> [RFU/area/min]	σ(α_p_) [RFU/area/min]
P*_E_-araE*	0.2%	4.0	2.9	77	22
	0.05%	14	7.2	92	23
	0.01%	32	12	57	26
P*_lac_-araE*	0.2%	1.5	1.9	45	19
	0.01%	1.6	2.0	20	9
	0.002%	2.3	3.8	11	6
	0.001%	1.7	4.2	5	4

Finally, we also analyzed the data of Fig. 5 with the full model of Fig. 4. We fitted the model by varying the basal transcription rate of arabinose uptake genes, the maximal expression rate of *gfp* and the arabinose export rate *k*, as above. To account for the different external arabinose concentrations in our experiments, we scaled the effective arabinose uptake velocity, V_upt_, according to Michaelis-Menten kinetics as described in Text S1. The model yields useful fits for both strains and all concentrations tested, as illustrated by a few representative fits in Fig. 5. For the reference strain, the resulting distributions of fit parameters indicate that all single-cell trajectories are compatible with a single underlying AraE uptake protein distribution at the time of sugar addition (Figs. S9–S11). Furthermore, they suggest that the Michaelis-Menten scaling of the arabinose uptake velocity with the external arabinose concentration consistently explains the observed modulation of the time delay. For the mutant with constitutive *araE* expression the resulting fit parameters display pronounced pairwise correlations (Figs. S12–S14). This is expected, since the slope of the fluorescence trajectories in this mutant is proportional to the product of basal uptake protein expression rate and *gfp* expression rate divided by the arabinose export rate. Hence, only a combination of the parameters is well constrained by the data.

## Discussion

We performed a detailed experimental and theoretical analysis of the single-cell switching dynamics in the arabinose utilization system of *E. coli*. In addition to traditional induction kinetics, in which cells are exposed to a step-like increase of the inducer (up-shift), we also measured, for the first time, the single-cell dynamics after sudden removal of arabinose (down-shift). Our up-shift data showed that a population of cells with functional arabinose catabolism displays significant, inducer-dependent heterogeneity in the onset of gene expression from the P*_BAD_* promoter. This observation indicates that the heterogeneous timing phenomenon of [23] is a genuine phenotype of the native arabinose system in *E. coli* cells. Our quantitative analysis is consistent with the interpretation that the timing heterogeneity is caused by a broad distribution of arabinose transporters at the time of induction, which is generated by the stochastic process of protein production at a low basal level. Our down-shift data shows rapid and homogeneous downregulation of gene expression, which does not depend on arabinose catabolism (Fig. 3). As the *ara* system is regulated by internal arabinose, this observation suggests another mechanism for arabinose depletion. Our quantitative model-based analysis indeed showed that a consistent description of all our data for the ‘off-to-on’ and ‘on-to-off’ transitions in several mutants requires the explicit incorporation of such a depletion mechanism.

Clearly, unnecessary protein expression is reduced by rapid and homogeneous downregulation of catabolic genes right after the external supply of substrate disappears. Therefore, the off-switching behavior of the arabinose system appears rational from a physiological perspective. It is less clear, however, why an additional depletion mechanism for internal arabinose is needed besides catabolism. Mechanistically, the depletion could rely on an efflux system. Toxic effects of high concentrations of sugars may have provided evolutionary pressure for the acquisition of such efflux systems [36]. Here, we pursued indications that arabinose export might be mediated by the *araJ* gene, which displays sequence homology to the MFS superfamily [24] and is annotated as a putative arabinose efflux transporter [25]. However, we did not find a significantly delayed shut-down of the arabinose system in a Δ*araJ* mutant compared to a strain without this deletion. Hence, we conclude that AraJ does not provide the primary depletion mechanism responsible for the rapid P*_BAD_* shut-down observed in our experiments. Arabinose might exit the cell via unspecific sugar exporters [37] or more specific sugar efflux transporters that have been shown to interfere with arabinose accumulation and the induction of the P*_BAD_* promoter [38], [39]. Alternatively, arabinose could exit via the uptake machinery itself: While arabinose import via AraFGH relies on ATP hydrolysis, such that the reverse reaction should be strongly suppressed, arabinose transport via AraE is only driven by the proton motive force. If cells are filled with arabinose in an arabinose-free medium, the strong outward-facing arabinose gradient can likely no longer be balanced by an inward-directed proton gradient, such that arabinose can leak out.

It is interesting to note that sugar export mechanisms have also been implied in other contexts. Growth inhibition may result from the uptake of toxic sugar analogues that inhibit uptake of bona fide sugar substrates or interfere with normal metabolism [37]. For instance, the non-metabolizable lactose analogue thiomethylgalactoside (TMG) is exported from *S. pyogenes* [40] and appears to be excreted from *E. coli* cells as well, since rapid switching from the on- to the off-state is observed in the *lac* operon when suddenly deprived of external TMG [19]. Moreover, upon addition of lactose to a strain with constitutively expressed *lac* operon, intermediates of lactose catabolism (glucose, galactose, and allolactose) rapidly accumulate in the medium [41] and it was suggested that sugar efflux is an integral part of the metabolism of lactose in *E. coli* [42]. In the case of the arabinose system, however, there is no apparent physiological advantage of arabinose export. Instead, an export mechanism might be useful for arabinose analogues, such as D-xylose and D-fucose: While these compounds are competitive inhibitors for L-arabinose uptake [31] and are thus likely to be imported by the arabinose permease system, their rate of metabolization via the arabinose isomerase AraA is greatly reduced [43]. Hence, in the absence of suitable export mechanisms such compounds might accumulate to toxic amounts.

The heterogeneous timing that we observed in the on-switching of the native arabinose system is qualitatively consistent with a previous study [22] that measured the onset of transcription from the P*_BAD_* promoter using single-RNA detection in live *E. coli* cells. On a quantitative level, the typical time delay of that study, from the medium shift until the first mRNA molecule could be detected, was even longer (∼50 min even at high arabinose concentrations) than the delay determined from our experiments. We attribute this quantitative difference to the fact that ref. [22] used a different strain and a different microfluidic setup, which likely leads to different physiological conditions. Taken together, we consider the previous [22], [23] and present work as convincing evidence that heterogeneous timing in the on-switching kinetics is a robust phenomenon of the arabinose system.

To test our quantitative understanding of the arabinose system, we also analyzed the single-cell expression kinetics of the P*_BAD_* promoter in a strain with constitutive arabinose transporter production. Such constructs are often used as gene expression systems [44], e.g., for biotechnological applications, when a controlled, homogeneous production of proteins in all cells of a culture is desired [34], [45], [46]. Similarly, we found that variation of external inducer concentration leads to a direct modulation of the protein production rate (Figs. 4 and 5). Our quantitative model provides a coherent and mechanistic explanation for the graded, dose-dependent response found in strains with constitutive *araE* expression [34], [45]. Taken together, our results shed new light on the regulatory dynamics of one of the best-studied systems in bacterial gene regulation and are useful for the redesign of cells in synthetic biology and biotechnological applications.

## Supporting Information

Figure S1 **Incremental doubling times of strains MG1655, BW25113 and JW0386-1 under pulsed (0–40 min) and continuous (0–100 min) arabinose supply for the same arabinose concentrations as in Fig. S3.** The doubling time was inferred for each time interval from the slopes of the single cell time traces of the cell area in phase contrast images. The plot shows the mean and standard error to the mean averaged of a population of cells under the respective condition.
(PDF)

Figure S2 **Correlations between GFP production rate α_p_ and delay time τ_d_ in strain MG1655.** Cells were induced with indicated concentrations of arabinose, and paremeters were inferred with the simple GFP production model published in ref. [23] of the main text.
(PDF)

Figure S3 **Control experiments of strains MG1655, BW25113 and JW0386-1 without arabinose down-shift.** Response kinetics of MG1655, BW25113, JW0386-1 to induction with arabinose and subsequent arabinose down-shift (A, C, E; data already shown with selected fits in Fig. 3 in the main text) compared to the respective control experiments without arabinose down-shift (B, D, F). In the control experiments fluorescence continuously increases, as expected. (*respective upper panels*) Black lines with symbols are experimental fluorescence trajectories and fits of the model dynamics [Eqs. (1)–(7)] under the appropriate conditions are shown as red lines. (*respective lower panels*) The corresponding dynamics of P*_BAD_* promoter activities in individual cells as inferred from the model. Note that the slightly smaller inducer concentration in the control experiments D and F leads to slightly longer response times. Number of evaluated cells: 62 (strain MG1655, 0–40 min), 19 (strain MG1655, control), 55 (strain BW25113, 40 min), 65 (strain BW25113, control), 51 (strain JW0386-1, 40 min), 51 (strain JW0386-1, control).
(PDF)

Figure S4 **Histograms of estimated parameters for the response kinetics of MG1655, BW25113, JW0386-1 in Fig. S3.** Grey shaded columns indicate parameters estimated from arabinose pulse experiments in Fig. S3A, C, and E (0–40 min arabinose) and white shaded columns indicate parameters estimated from experiments with continuous arabinose supply in Fig. S3B, D, and F (0–100 min arabinose). The top row shows the histograms of basal *araE* expression rates, the middle row shows the histograms of maximal *gfp* expression rates and the lower row shows the histograms of the arabinose export rates at indicated arabinose concentrations. Note that the arabinose loss rate *k* is only well constrained by our data in the case of pulsed arabinose addition (0–40 min arabinose) and in the case of continuous arabinose supply we found large variations of the estimated arabinose loss rates between individual cells of the population. In fact, in the latter case only combinations of parameters are well constrained by our data, as revealed by the scatter plots in Fig. S6.
(PDF)

Figure S5 **Scatter plots of the fit parameters in Fig. S4 versus the corresponding χ^2^-value.** Grey shaded columns indicate parameters estimated from arabinose pulse experiments in Fig. S3A, C, and E (0–40 min arabinose) and white shaded columns indicate parameters estimated from experiments with continuous arabinose supply in Fig. S3B, D, and F (0–100 min arabinose).
(PDF)

Figure S6 **Pairwise scatter plots of individual fit parameters of Fig. S4 against each other.** Grey shaded columns indicate parameters estimated from arabinose pulse experiments in Fig. S3A, C, and E (0–40 min arabinose) and white shaded columns indicate parameters estimated from experiments with continuous arabinose supply in Fig. S3B, D, and F (0–100 min arabinose).
(PDF)

Figure S7 **Derivation of the delay time distribution in a model with arabinose export.** (A) Theoretical delay time as a function of the initial number of transporter proteins AraE. The delay time was defined as the time until the P_BAD_ promoter reached 95% of its maximal activity and was estimated from numerical simulations of our model [Eqs. (1)–(7) in the main text] under the initial addition of indicated levels of external arabinose. For all arabinose concentrations we observe a monotonic decrease of the delay time with increasing transporter levels, but with a characteristic kink at around 500 AraE molecules/cell. This strong decrease of the delay time occurs in a regime, in which the high initial number of AraE molecules results in an internal arabinose (quasi-)steady-state level that is already high enough to activate the promoter. For AraE numbers below this kink, the delay time scales to a first approximation inversely with the number of AraE molecules (dotted line). The detailed shape of the curves, however, depends on the nature of the nonlinear feedback of AraE on its own synthesis, and we find that the delay time increases stronger than the 1/N-scaling at low AraE numbers. Theoretical delay time distributions (B) and cumulative probabilities (C) at different external arabinose concentrations in our model with arabinose efflux. These data were obtained by using the relation in (A) to transform a negative binomial distribution of arabinose transporters at the time of sugar addition, P(n), into its corresponding delay time distribution Q(τ_d_), analogous to (Megerle et al., 2008). To that end, we numerically applied the transformation rule Q(τ_d_) = |dn(τ_d_)/dτ_d_ |P(n) and used <n> = 110 and σ_n_ = 60 as parameters for P(n). Due to the inverse scaling of the delay time with the number of AraE proteins on the support of P(n) in (A), the delay time distributions in obtained here (B; colored lines) only differ within experimental error from our previous results for the delay time distribution in a model without arabinose efflux (B; black solid line).
(PDF)

Figure S8 **Theoretical expression kinetics in an alternative model with arabinose export via AraE.** The model reproduces the main features of the experimental data reported in this study: (A) Heterogeneous timing of gene induction; (B) Rapid shut-down of transcription after arabinose removal; (C) Modulation of expression rate with external arabinose concentration in a model with constitutive *araE* expression. The parameters of the alternative model were chosen as in the model of the main text (see Table S2), with the following exceptions: V_max_ = 120 molecules protein^−1^ min^−1^, *K_m_* = 2.8 mM, *k* = 3×10^−4^ min^−1^ protein^−1^. To illustrate stochastic variability in the initial number of uptake proteins, trajectories are shown in (A) and (B) for 400, 300, 200 and 100 AraE molecules at each arabinose concentration. In (C) the initial numbers of AraE molecules was set to 10 times higher values, corresponding to an elevated basal expression level of P*_lac_* compared to P*_E_.*
(PDF)

Figure S9 **Histograms of estimated parameters in the reference strain JW1889-3.** The top row shows the histograms of basal *araE* expression rates, the middle row shows the histograms of maximal *gfp* expression rates and the lower row shows the histograms of the arabinose export rates at indicated arabinose concentrations.
(PDF)

Figure S10 **Scatter plots of the fit parameters in Fig. S9 versus the corresponding χ^2^-value.**
(PDF)

Figure S11 **Pairwise scatter plots of individual fit parameters of Fig. S9 against each other.**
(PDF)

Figure S12 **Histograms of estimated parameters in the reference strain JW1889-5.** The top row shows the histograms of basal *araE* expression rates, the middle row shows the histograms of maximal *gfp* expression rates and the lower row shows the histograms of the arabinose export rates at indicated arabinose concentrations.
(PDF)

Figure S13 **Scatter plots of the fit parameters in Fig. S12 versus the corresponding χ^2^-value.**
(PDF)

Figure S14 **Pairwise scatter plots of individual fit parameters of Fig. S12 against each other.**
(PDF)

Table S1 **Bacterial strains used in this study.**
(DOC)

Table S2 **Parameters used in the mathematical model.**
(DOC)

Table S3 **Statistics of fitted parameters in Fig. S4.**
(DOCX)

Text S1 **Construction of bacterial strains and discussion of modeling rationale.**
(DOC)
