# Supplementary figures and images for: Single Cell Kinetics of Phenotypic Switching in the Arabinose Utilization System of E. coli
Source: PLoS One. 2014 Feb 26;9(2):e89532. doi: 10.1371/journal.pone.0089532 (PMC3935871; doi:10.1371/journal.pone.0089532)

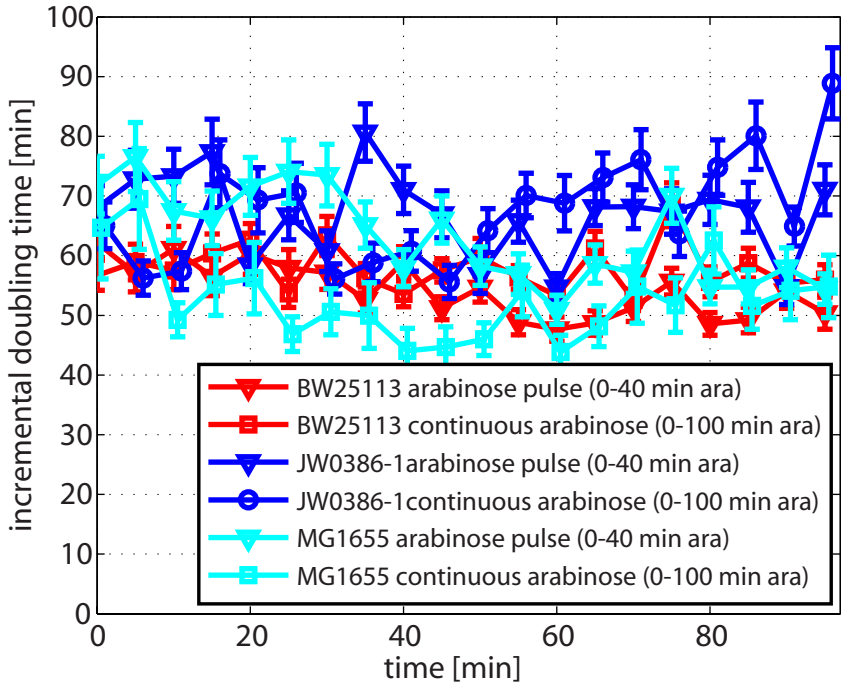

Supplement: Figure S1 — Incremental doubling times of strains MG1655, BW25113 and JW0386-1 under pulsed (0–40 min) and continuous (0–100 min) arabinose supply for the same arabinose concentrations as in Fig. S3. The doubling time was inferred for each time interval from the slopes of the single cell time traces of the cell area in phase contrast images. The plot shows the mean and standard error to the mean averaged of a population of cells under the respective condition. (PDF) [file pone.0089532.s001.pdf]

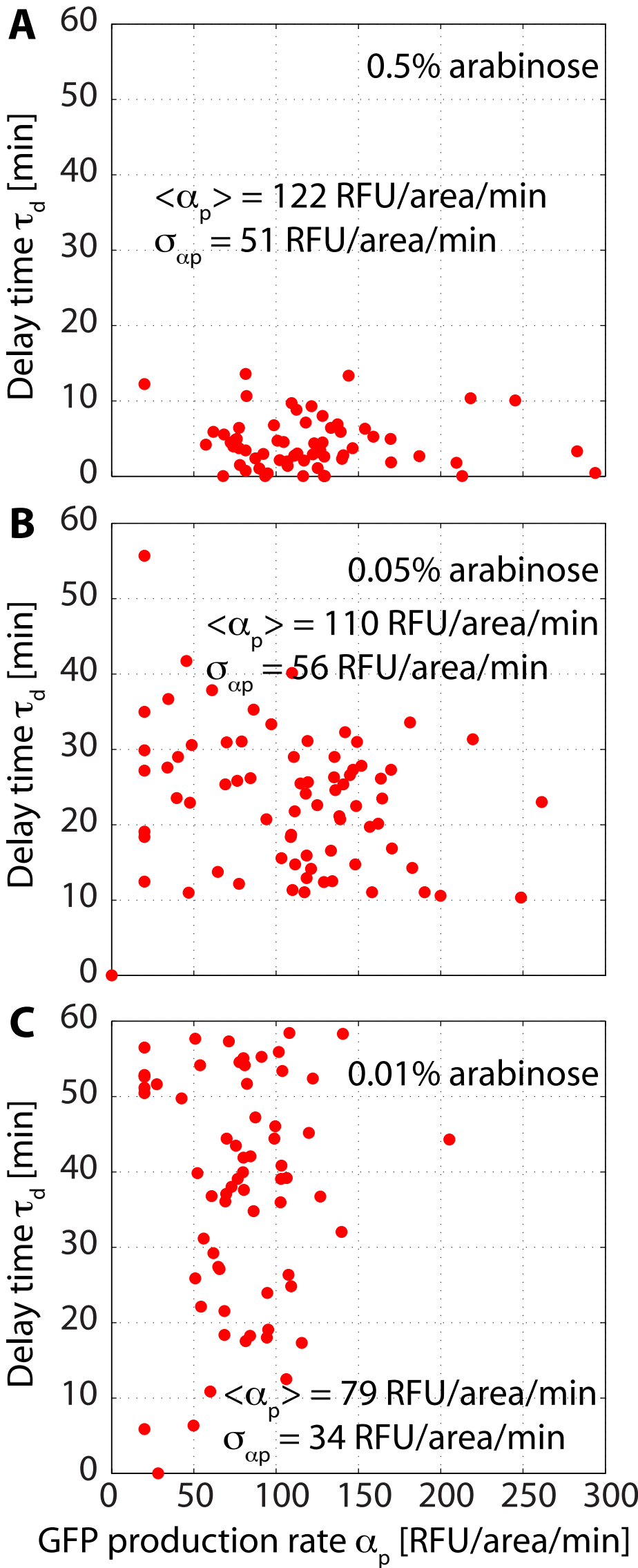

Supplement: Figure S2 — Correlations between GFP production rate αp and delay time τd in strain MG1655. Cells were induced with indicated concentrations of arabinose, and paremeters were inferred with the simple GFP production model published in ref. [23] of the main text. (PDF) [file pone.0089532.s002.pdf]

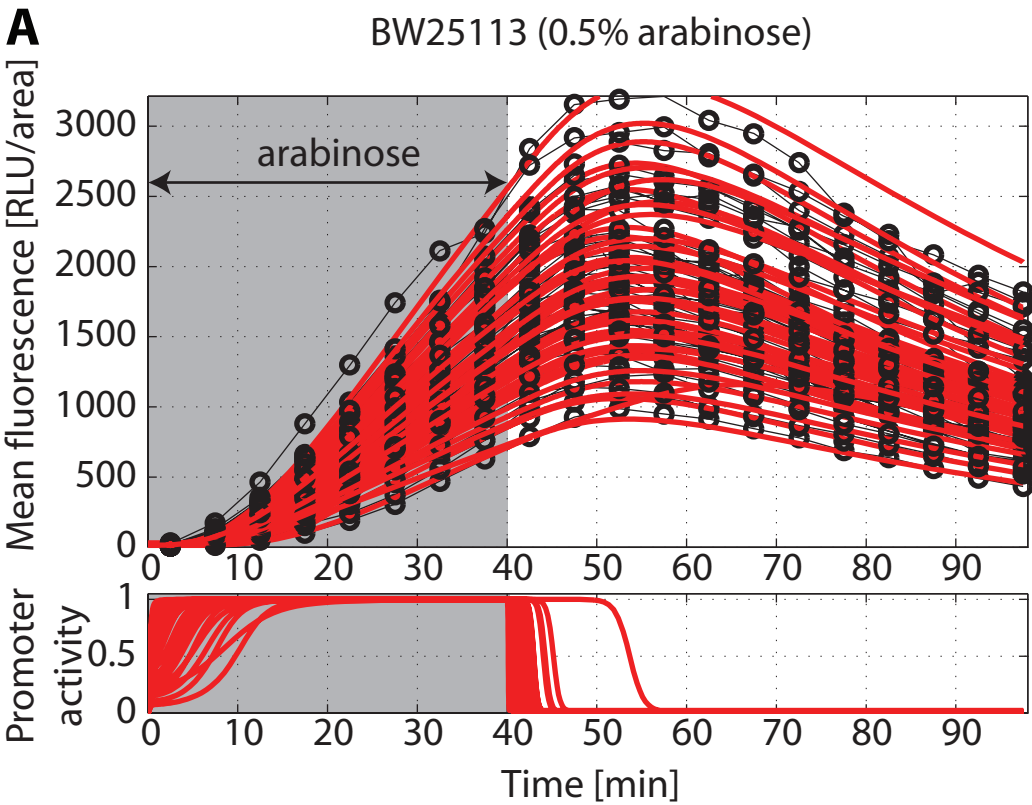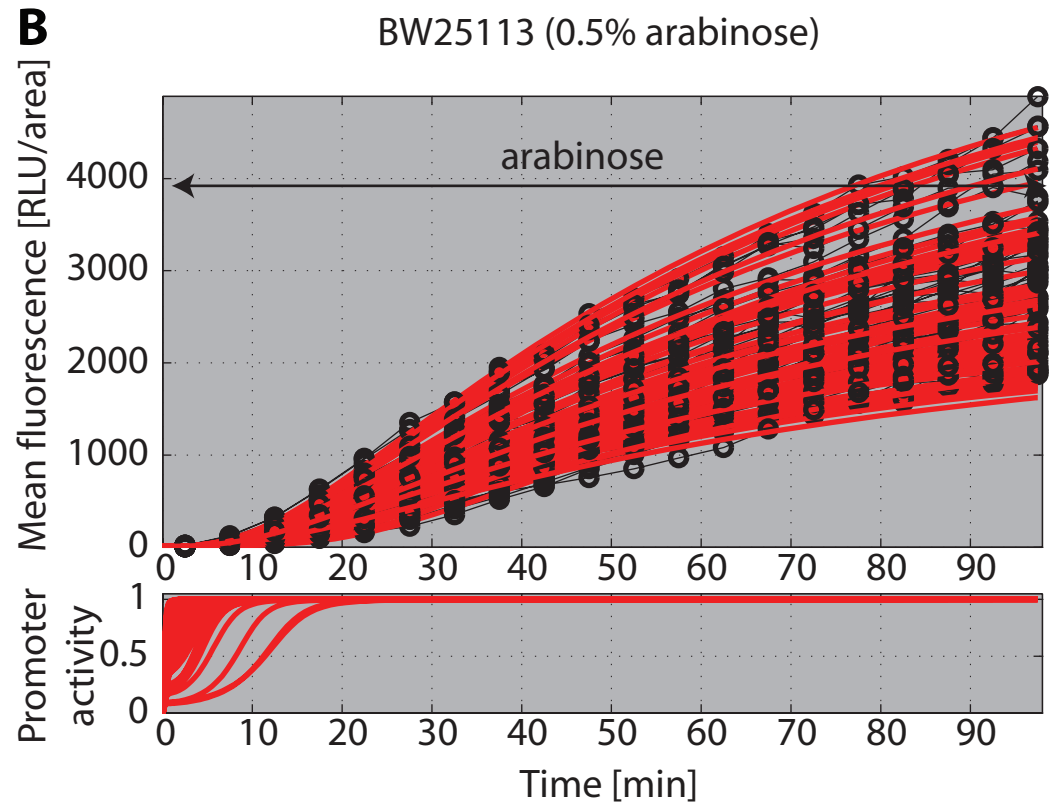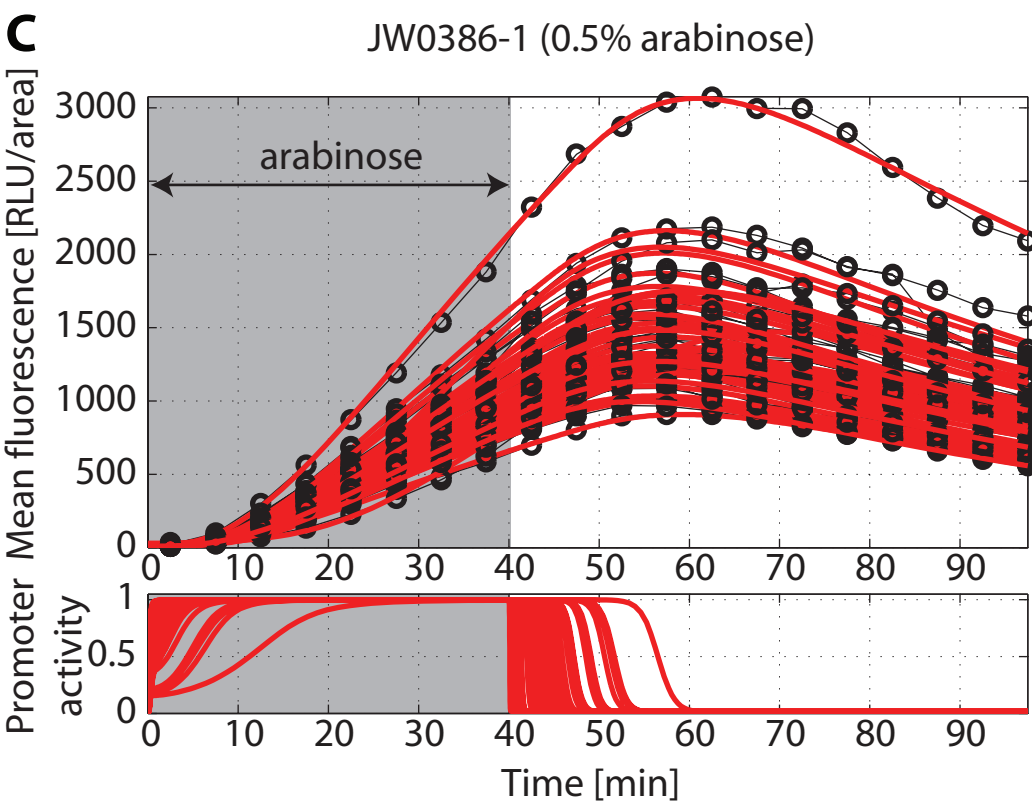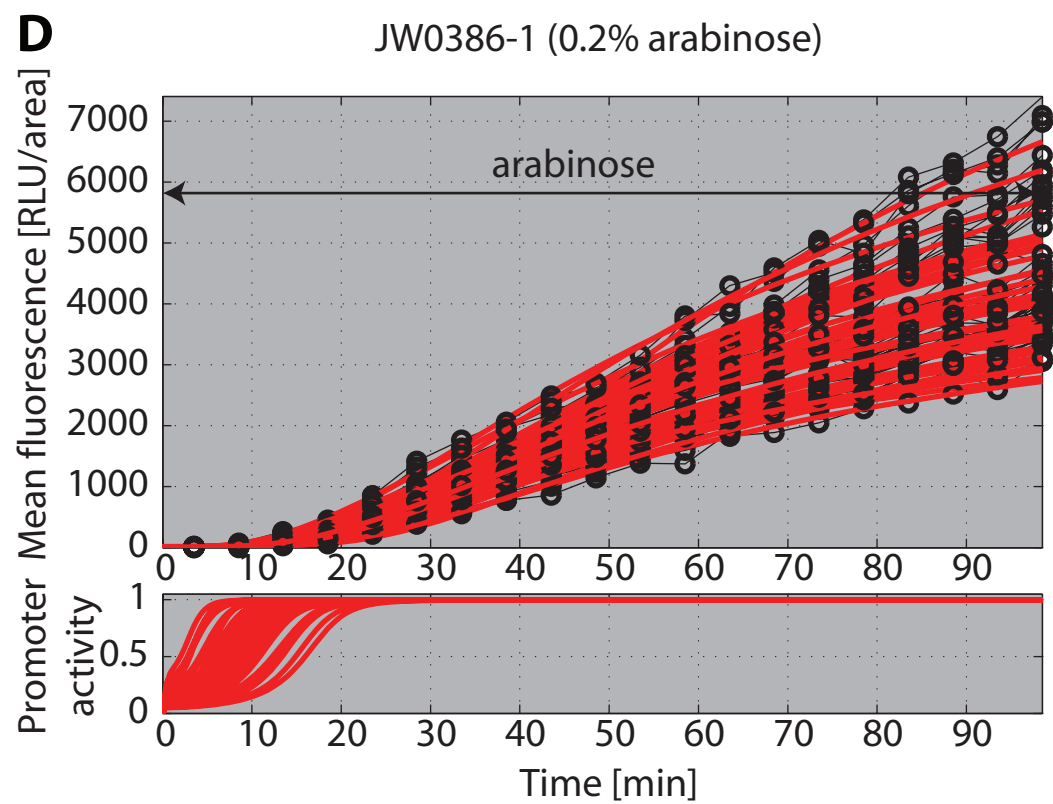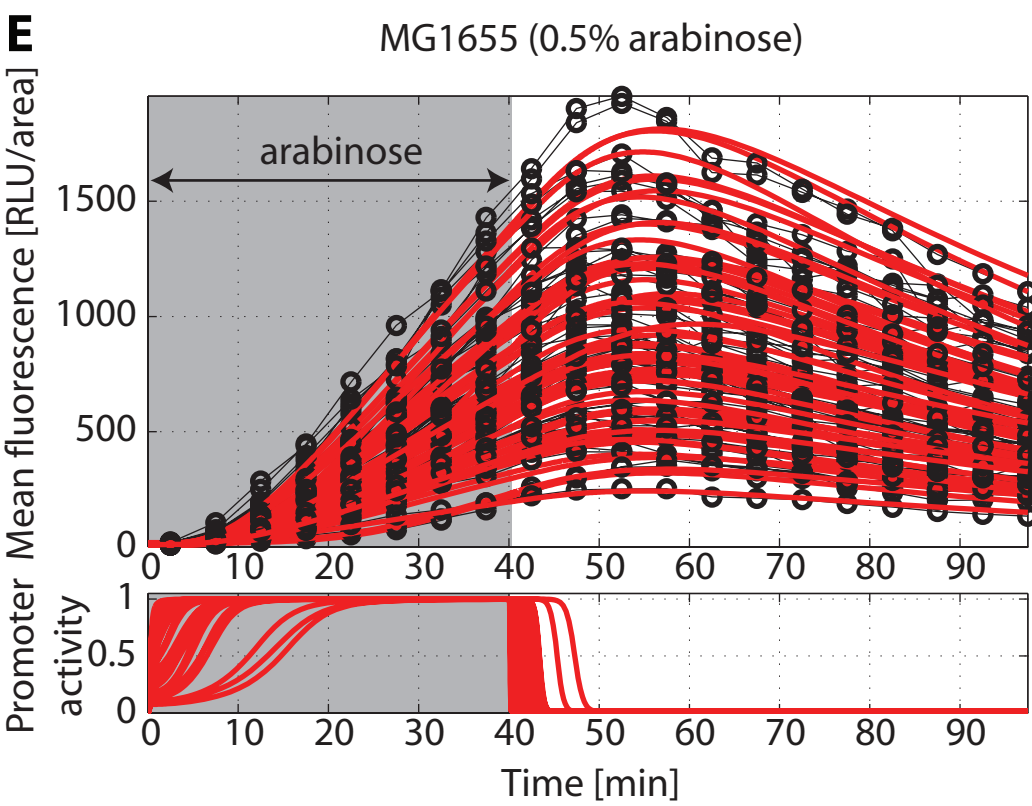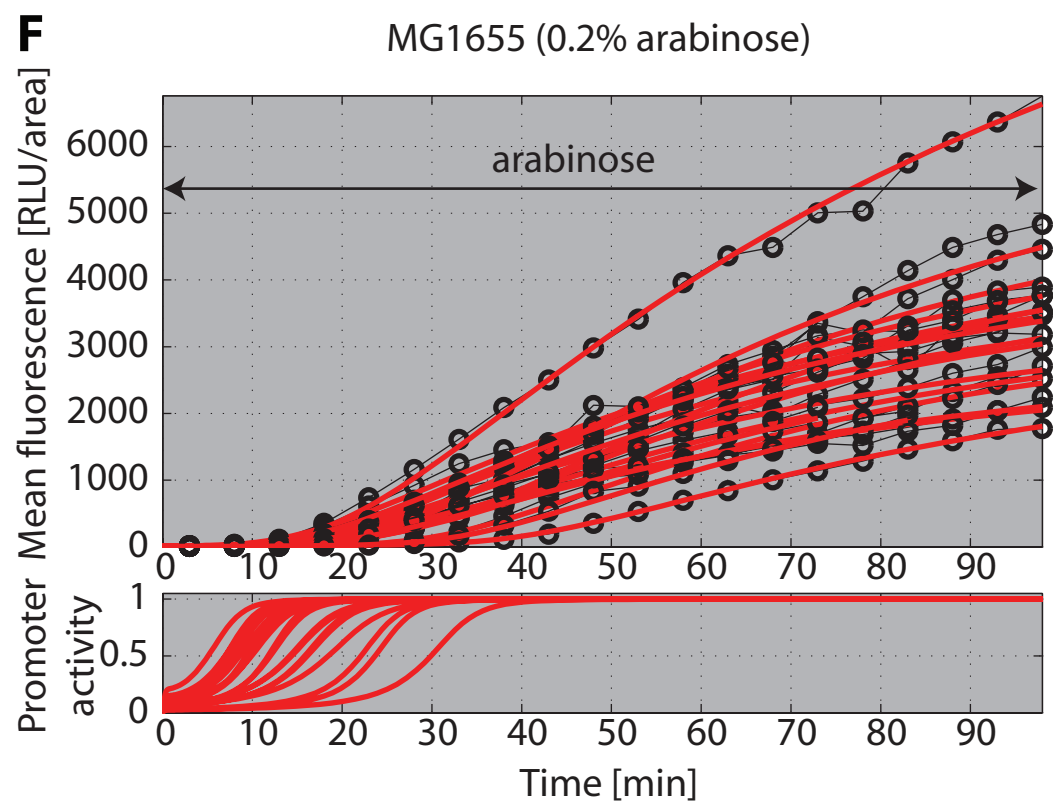

Supplement: Figure S3 — Control experiments of strains MG1655, BW25113 and JW0386-1 without arabinose down-shift. Response kinetics of MG1655, BW25113, JW0386-1 to induction with arabinose and subsequent arabinose down-shift (A, C, E; data already shown with selected fits in Fig. 3 in the main text) compared to the respective control experiments without arabinose down-shift (B, D, F). In the control experiments fluorescence continuously increases, as expected. (respective upper panels) Black lines with symbols are experimental fluorescence trajectories and fits of the model dynamics [Eqs. (1)–(7)] under the appropriate conditions are shown as red lines. (respective lower panels) The corresponding dynamics of PBAD promoter activities in individual cells as inferred from the model. Note that the slightly smaller inducer concentration in the control experiments D and F leads to slightly longer response times. Number of evaluated cells: 62 (strain MG1655, 0–40 min), 19 (strain MG1655, control), 55 (strain BW25113, 40 min), 65 (strain BW25113, control), 51 (strain JW0386-1, 40 min), 51 (strain JW0386-1, control). (PDF) [file pone.0089532.s003.pdf]

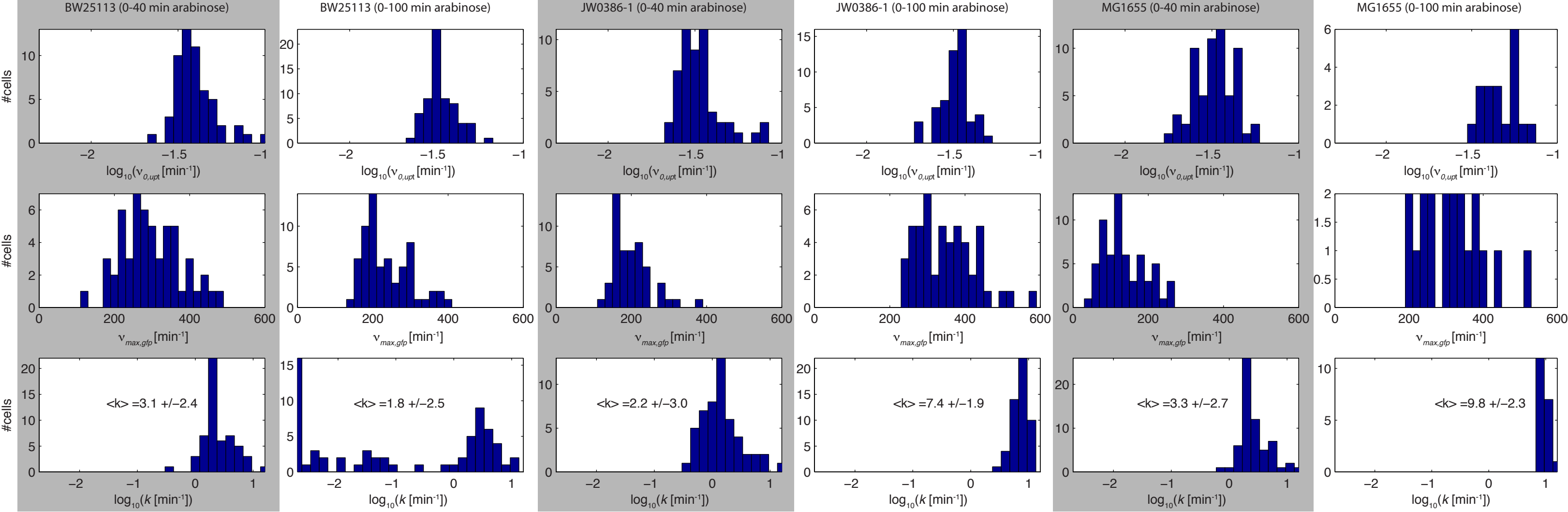

Supplement: Figure S4 — Histograms of estimated parameters for the response kinetics of MG1655, BW25113, JW0386-1 in Fig. S3. Grey shaded columns indicate parameters estimated from arabinose pulse experiments in Fig. S3A, C, and E (0–40 min arabinose) and white shaded columns indicate parameters estimated from experiments with continuous arabinose supply in Fig. S3B, D, and F (0–100 min arabinose). The top row shows the histograms of basal araE expression rates, the middle row shows the histograms of maximal gfp expression rates and the lower row shows the histograms of the arabinose export rates at indicated arabinose concentrations. Note that the arabinose loss rate k is only well constrained by our data in the case of pulsed arabinose addition (0–40 min arabinose) and in the case of continuous arabinose supply we found large variations of the estimated arabinose loss rates between individual cells of the population. In fact, in the latter case only combinations of parameters are well constrained by our data, as revealed by the scatter plots in Fig. S6. (PDF) [file pone.0089532.s004.pdf]

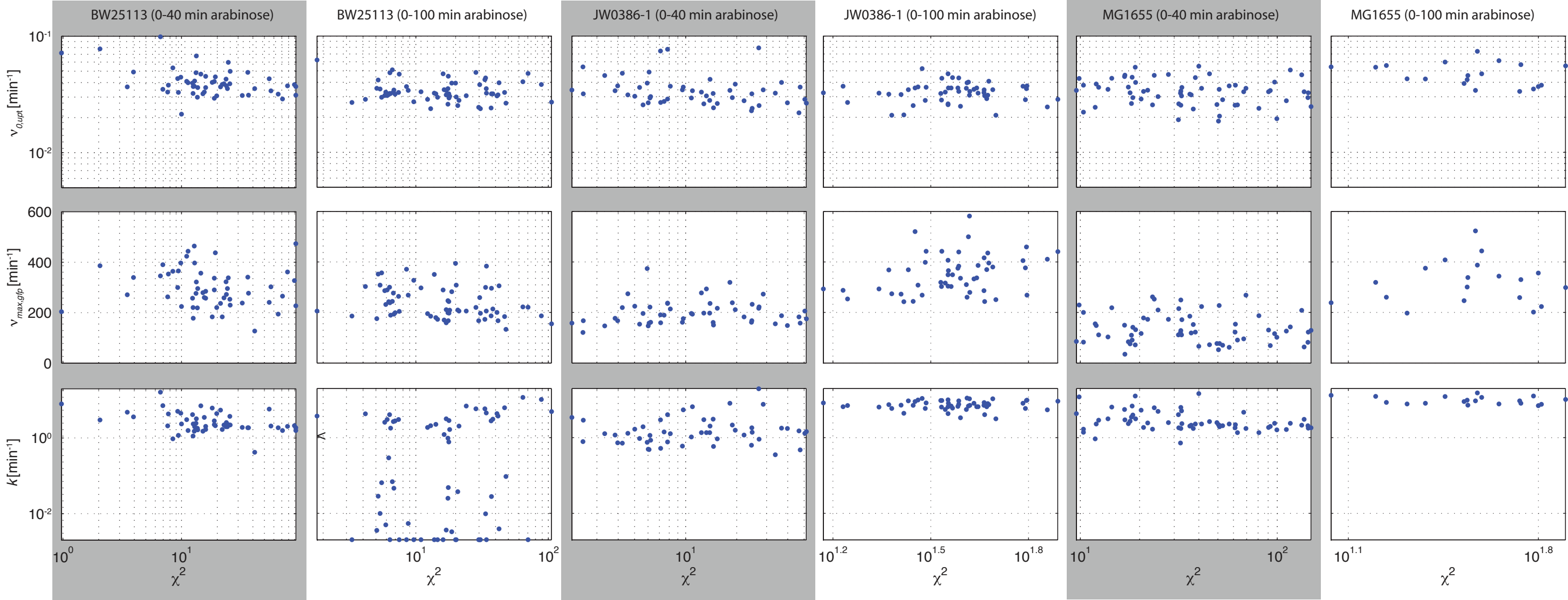

Supplement: Figure S5 — Scatter plots of the fit parameters in Fig. S4 versus the corresponding χ2-value. Grey shaded columns indicate parameters estimated from arabinose pulse experiments in Fig. S3A, C, and E (0–40 min arabinose) and white shaded columns indicate parameters estimated from experiments with continuous arabinose supply in Fig. S3B, D, and F (0–100 min arabinose). (PDF) [file pone.0089532.s005.pdf]

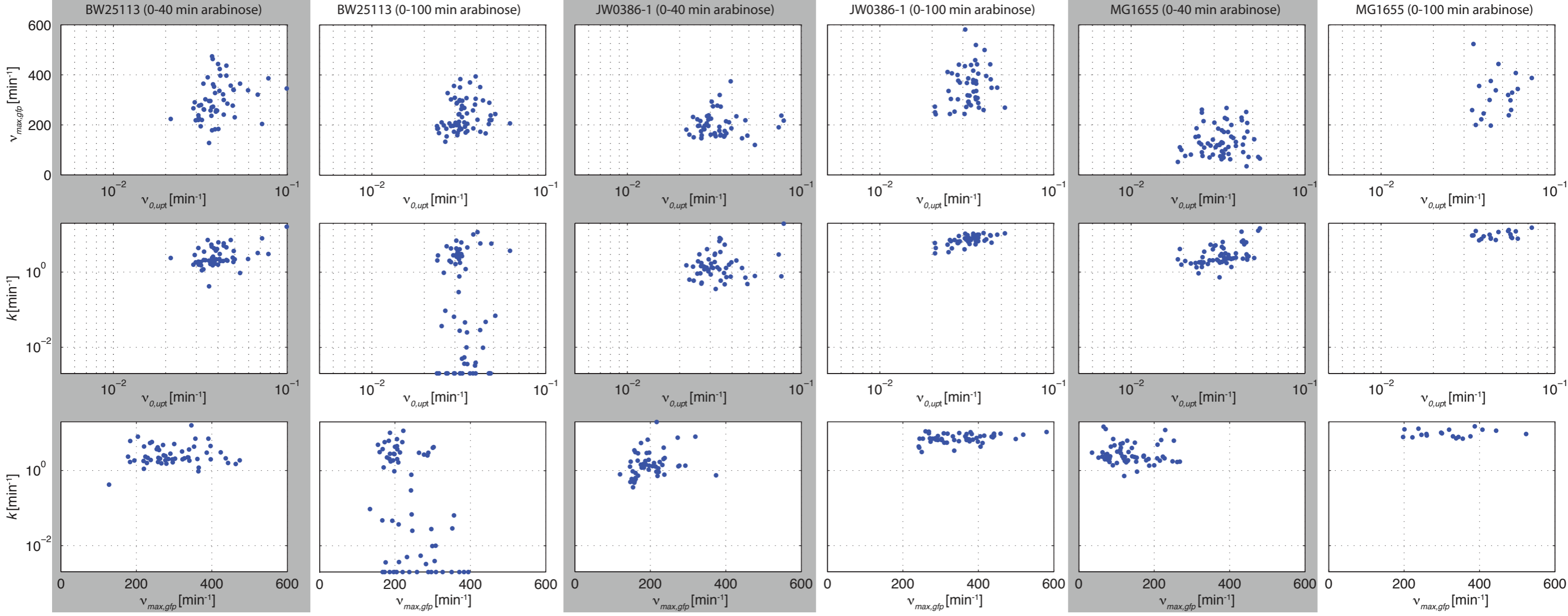

Supplement: Figure S6 — Pairwise scatter plots of individual fit parameters of Fig. S4 against each other. Grey shaded columns indicate parameters estimated from arabinose pulse experiments in Fig. S3A, C, and E (0–40 min arabinose) and white shaded columns indicate parameters estimated from experiments with continuous arabinose supply in Fig. S3B, D, and F (0–100 min arabinose). (PDF) [file pone.0089532.s006.pdf]

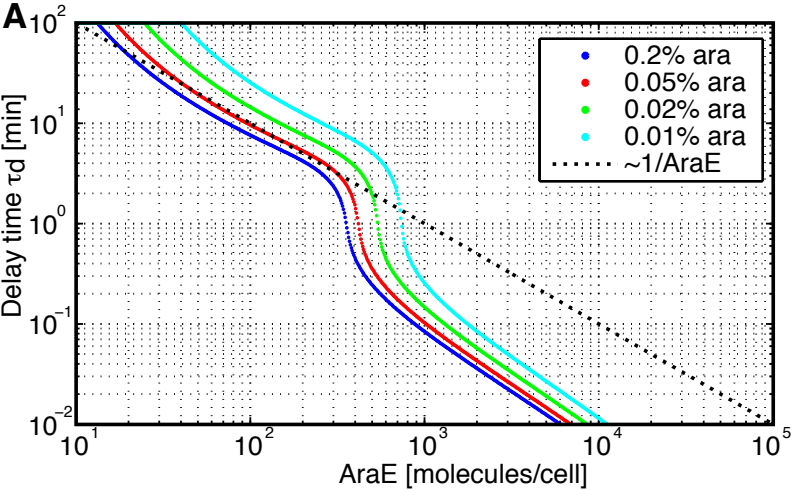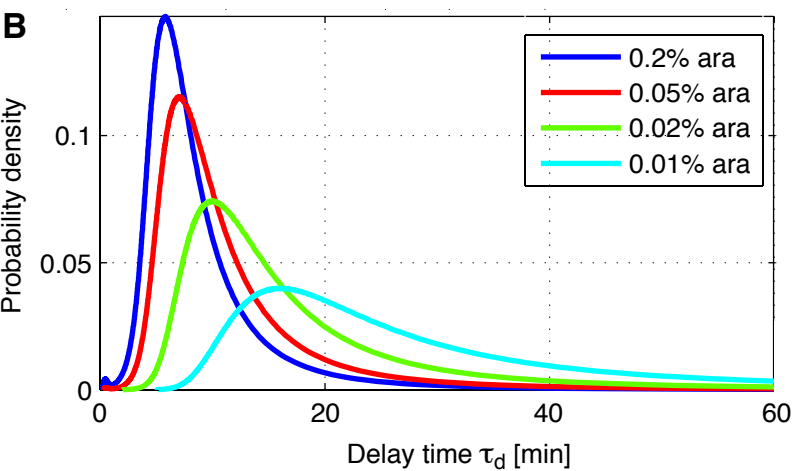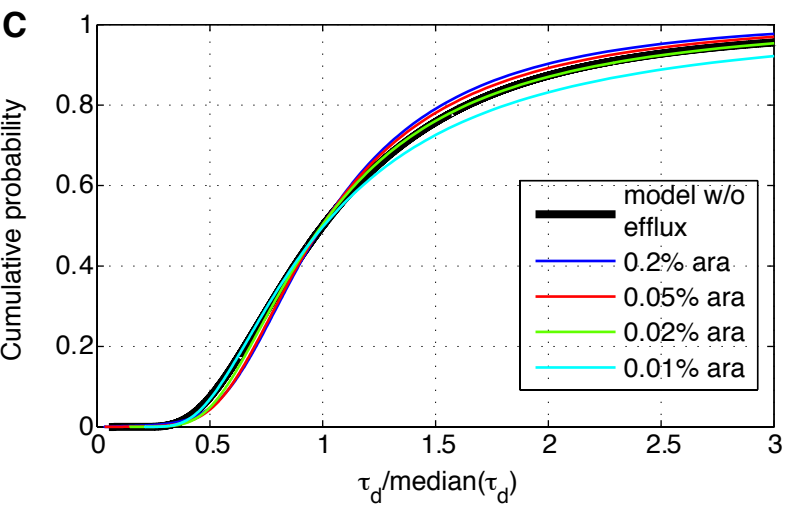

Supplement: Figure S7 — Derivation of the delay time distribution in a model with arabinose export. (A) Theoretical delay time as a function of the initial number of transporter proteins AraE. The delay time was defined as the time until the PBAD promoter reached 95% of its maximal activity and was estimated from numerical simulations of our model [Eqs. (1)–(7) in the main text] under the initial addition of indicated levels of external arabinose. For all arabinose concentrations we observe a monotonic decrease of the delay time with increasing transporter levels, but with a characteristic kink at around 500 AraE molecules/cell. This strong decrease of the delay time occurs in a regime, in which the high initial number of AraE molecules results in an internal arabinose (quasi-)steady-state level that is already high enough to activate the promoter. For AraE numbers below this kink, the delay time scales to a first approximation inversely with the number of AraE molecules (dotted line). The detailed shape of the curves, however, depends on the nature of the nonlinear feedback of AraE on its own synthesis, and we find that the delay time increases stronger than the 1/N-scaling at low AraE numbers. Theoretical delay time distributions (B) and cumulative probabilities (C) at different external arabinose concentrations in our model with arabinose efflux. These data were obtained by using the relation in (A) to transform a negative binomial distribution of arabinose transporters at the time of sugar addition, P(n), into its corresponding delay time distribution Q(τd), analogous to (Megerle et al., 2008). To that end, we numerically applied the transformation rule Q(τd) = |dn(τd)/dτd |P(n) and used = 110 and σn = 60 as parameters for P(n). Due to the inverse scaling of the delay time with the number of AraE proteins on the support of P(n) in (A), the delay time distributions in obtained here (B; colored lines) only differ within experimental error from our previous results for the delay time [file pone.0089532.s007.pdf]

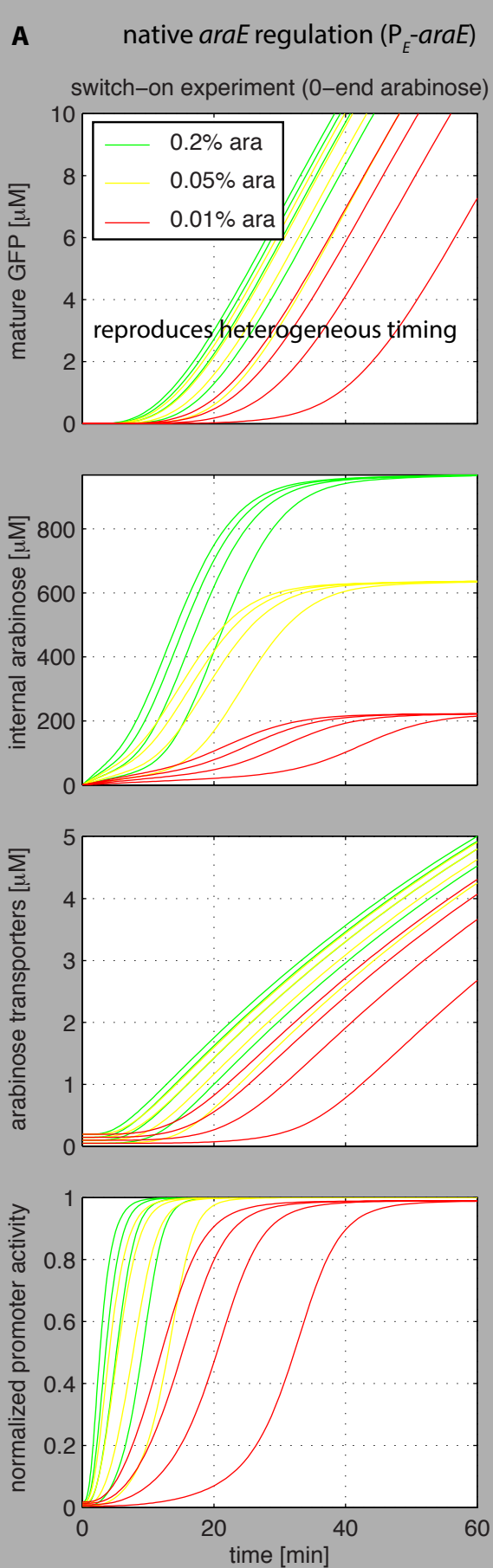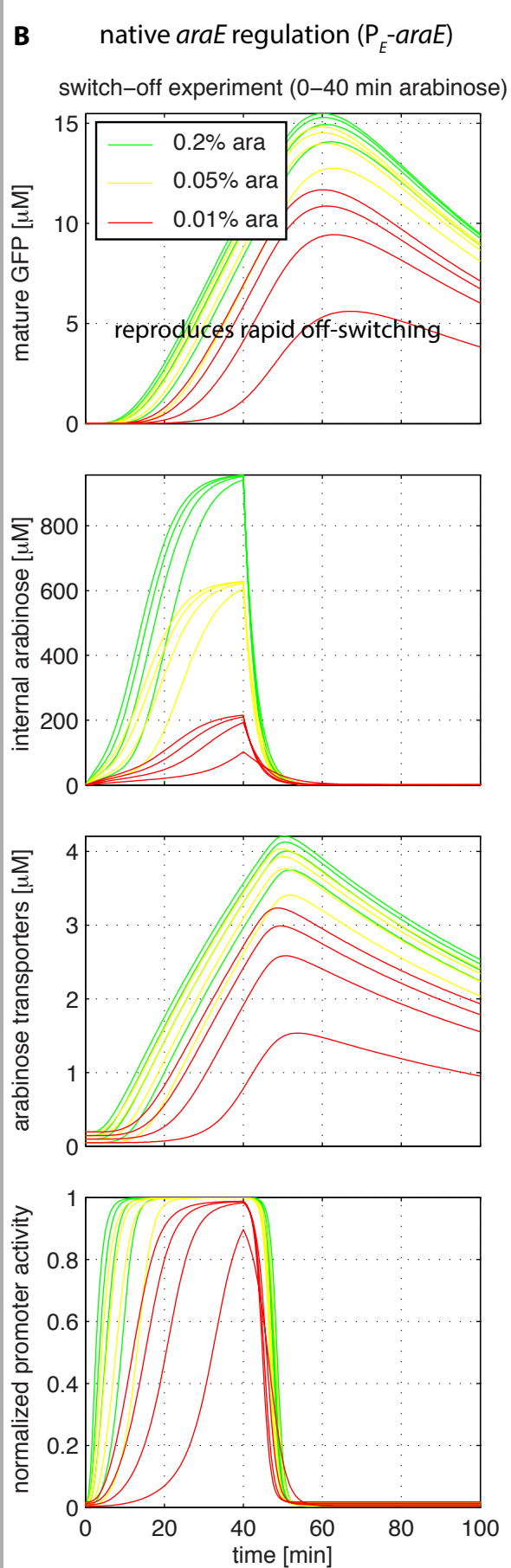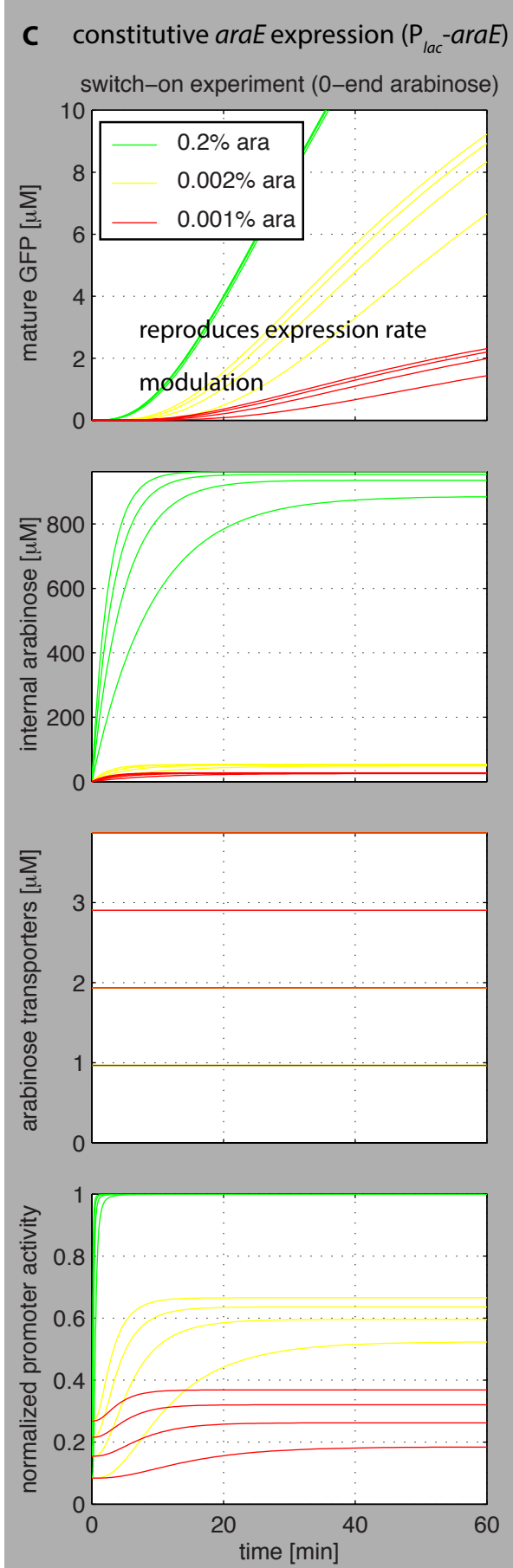

Supplement: Figure S8 — Theoretical expression kinetics in an alternative model with arabinose export via AraE. The model reproduces the main features of the experimental data reported in this study: (A) Heterogeneous timing of gene induction; (B) Rapid shut-down of transcription after arabinose removal; (C) Modulation of expression rate with external arabinose concentration in a model with constitutive araE expression. The parameters of the alternative model were chosen as in the model of the main text (see Table S2), with the following exceptions: Vmax = 120 molecules protein−1 min−1, Km = 2.8 mM, k = 3×10−4 min−1 protein−1. To illustrate stochastic variability in the initial number of uptake proteins, trajectories are shown in (A) and (B) for 400, 300, 200 and 100 AraE molecules at each arabinose concentration. In (C) the initial numbers of AraE molecules was set to 10 times higher values, corresponding to an elevated basal expression level of Plac compared to PE. (PDF) [file pone.0089532.s008.pdf]

0.2 % arabinose

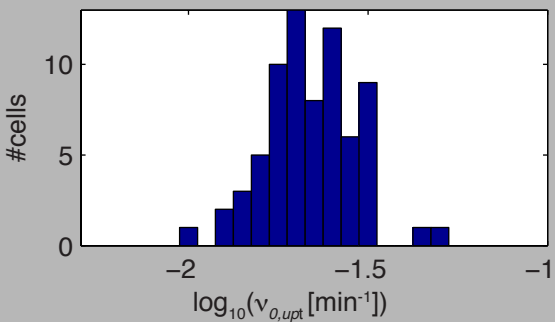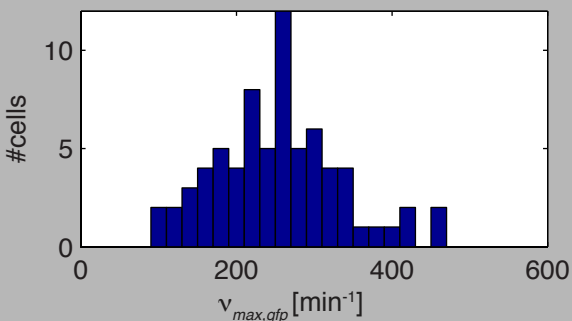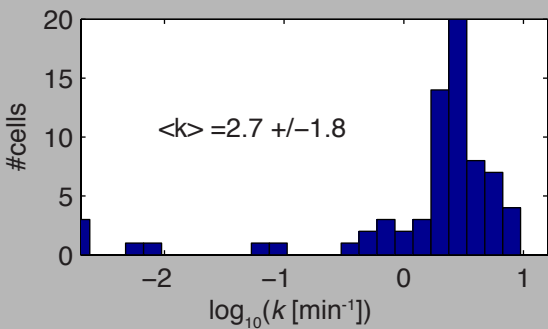

0.05 % arabinose

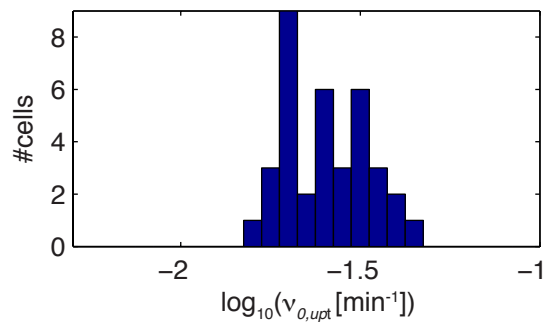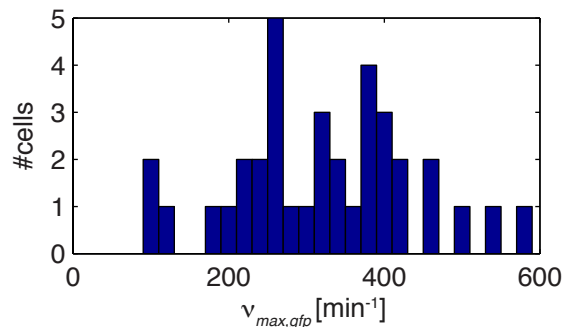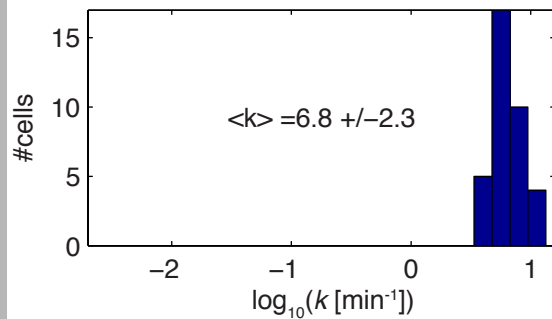

0.01 % arabinose

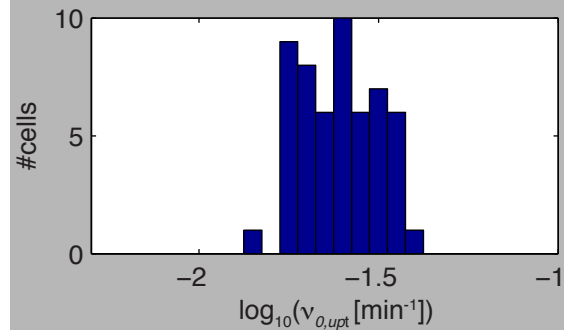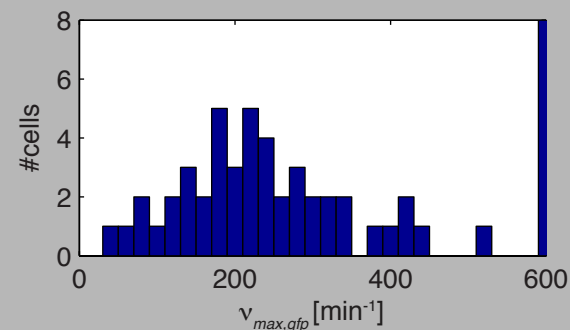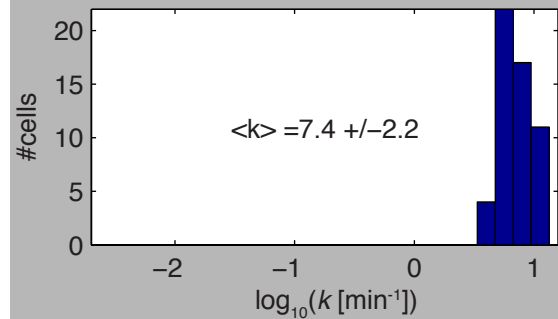

Supplement: Figure S9 — Histograms of estimated parameters in the reference strain JW1889-3. The top row shows the histograms of basal araE expression rates, the middle row shows the histograms of maximal gfp expression rates and the lower row shows the histograms of the arabinose export rates at indicated arabinose concentrations. (PDF) [file pone.0089532.s009.pdf]

0.2 % arabinose

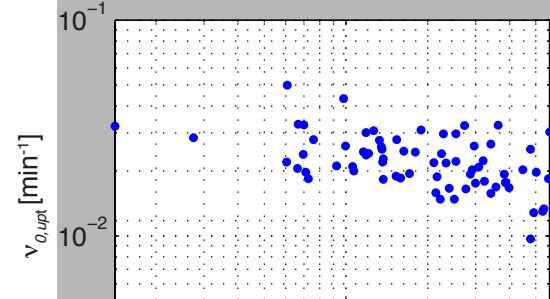

0.05 % arabinose

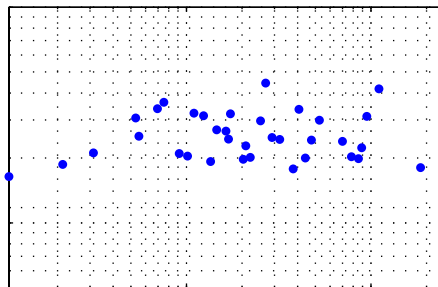

0.01 % arabinose

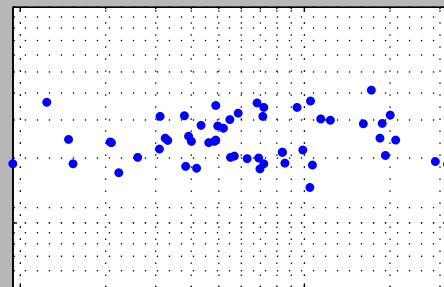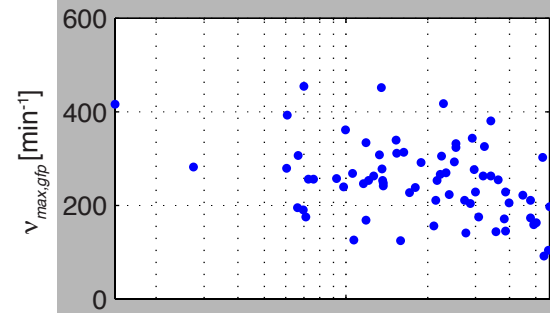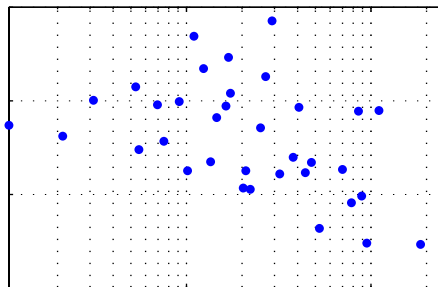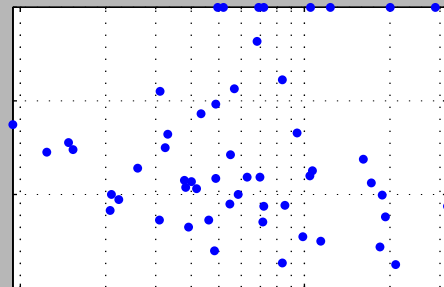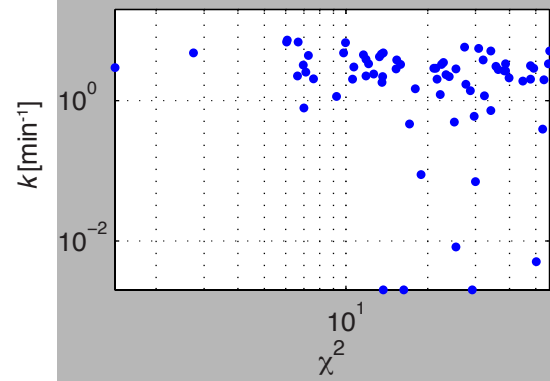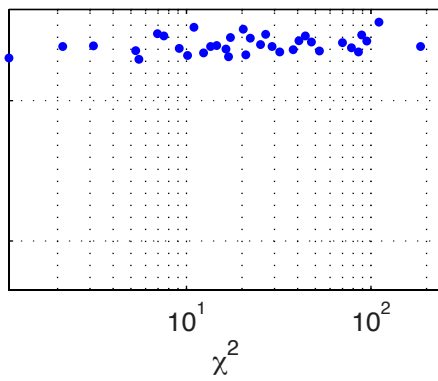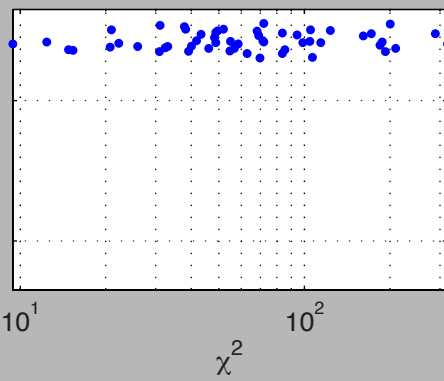

Supplement: Figure S10 — Scatter plots of the fit parameters in Fig. S9 versus the corresponding χ2-value. (PDF) [file pone.0089532.s010.pdf]

0.2 % arabinose

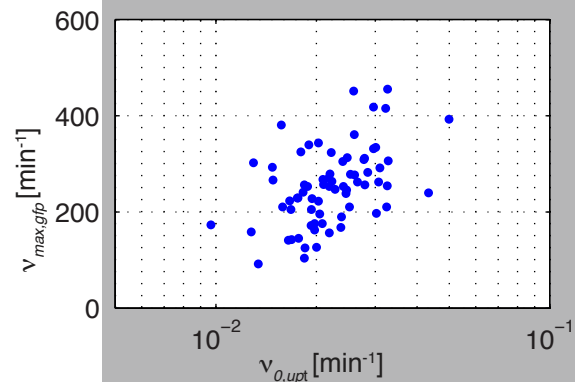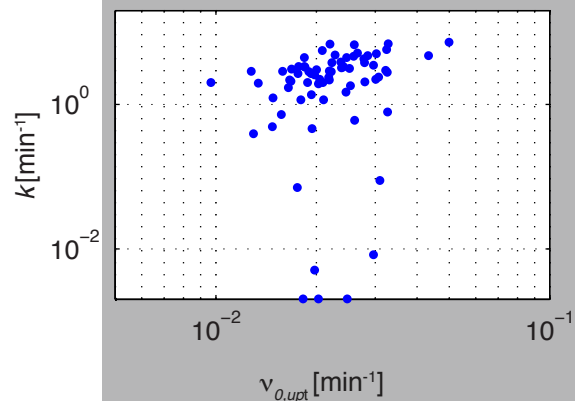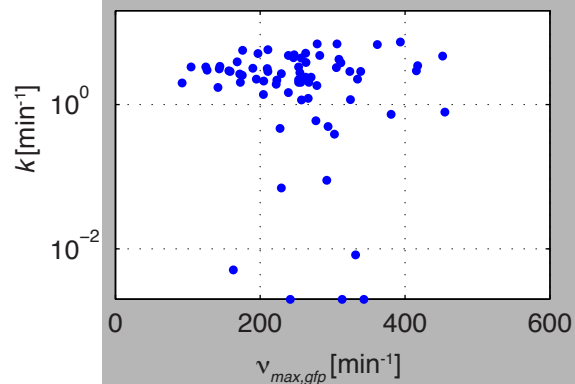

0.05 % arabinose

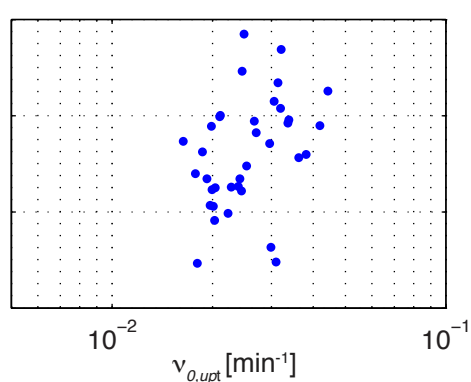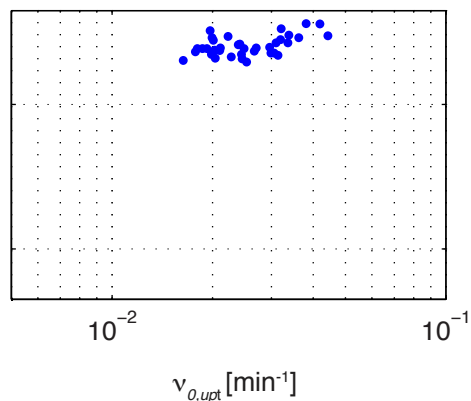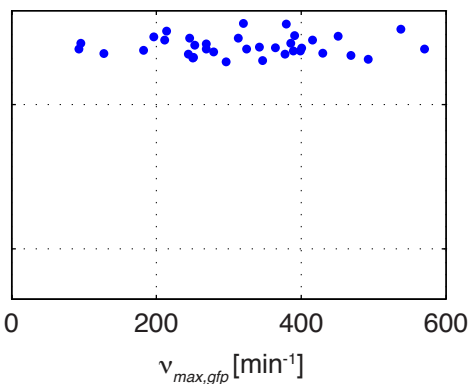

0.01 % arabinose

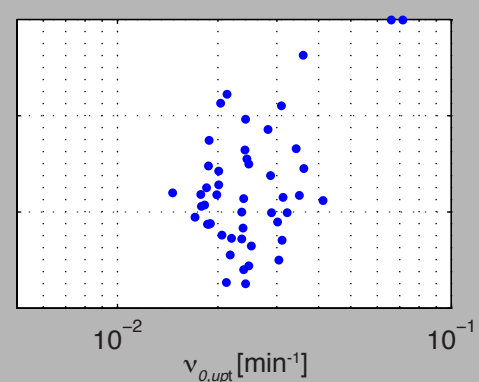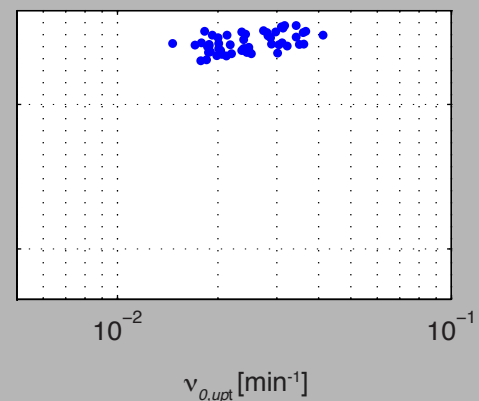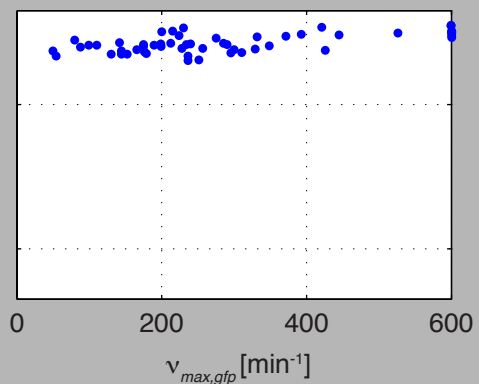

Supplement: Figure S11 — Pairwise scatter plots of individual fit parameters of Fig. S9 against each other. (PDF) [file pone.0089532.s011.pdf]

0.2 % arabinose

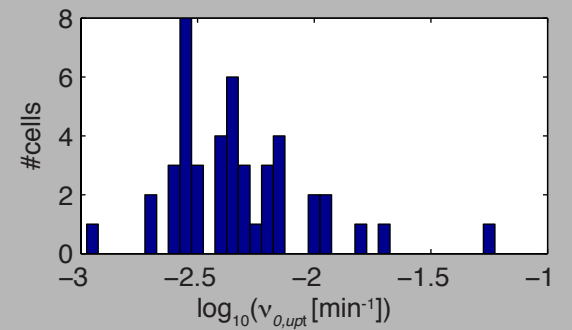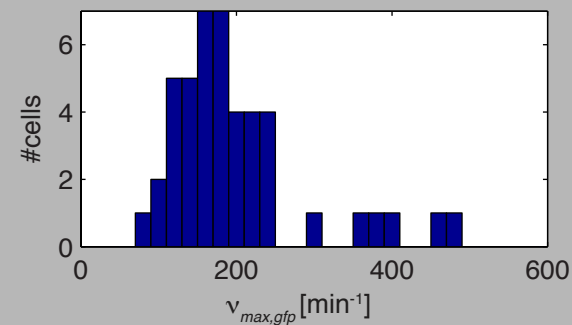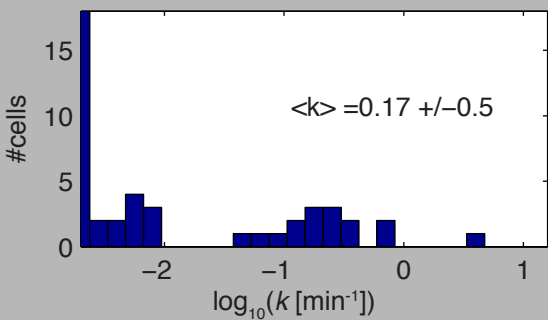

0.002 % arabinose

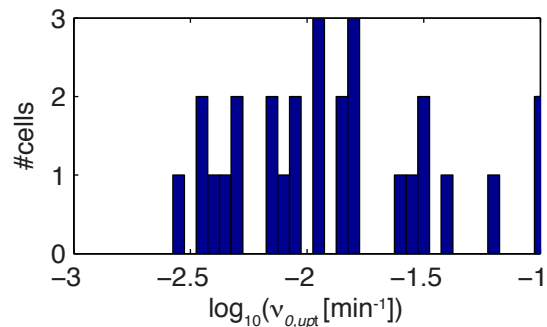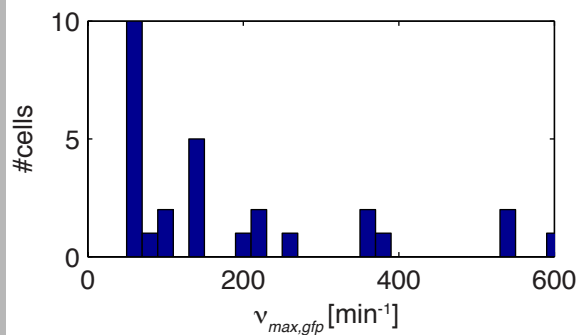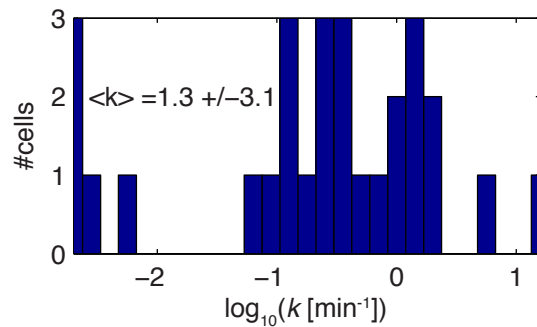

0.001 % arabinose

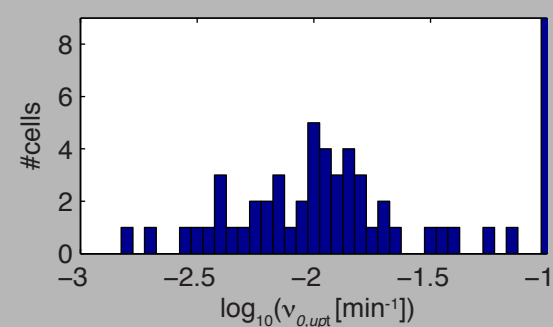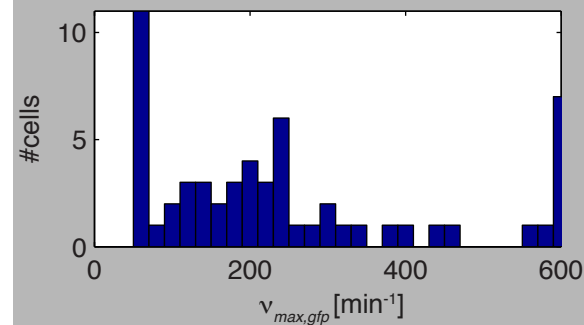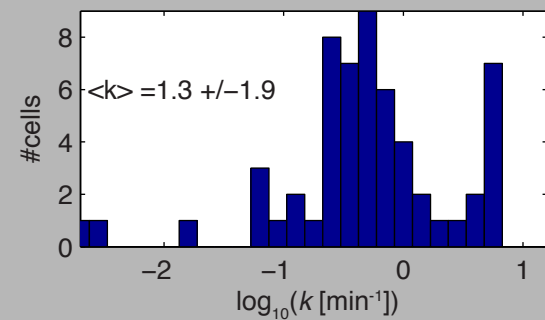

Supplement: Figure S12 — Histograms of estimated parameters in the reference strain JW1889-5. The top row shows the histograms of basal araE expression rates, the middle row shows the histograms of maximal gfp expression rates and the lower row shows the histograms of the arabinose export rates at indicated arabinose concentrations. (PDF) [file pone.0089532.s012.pdf]

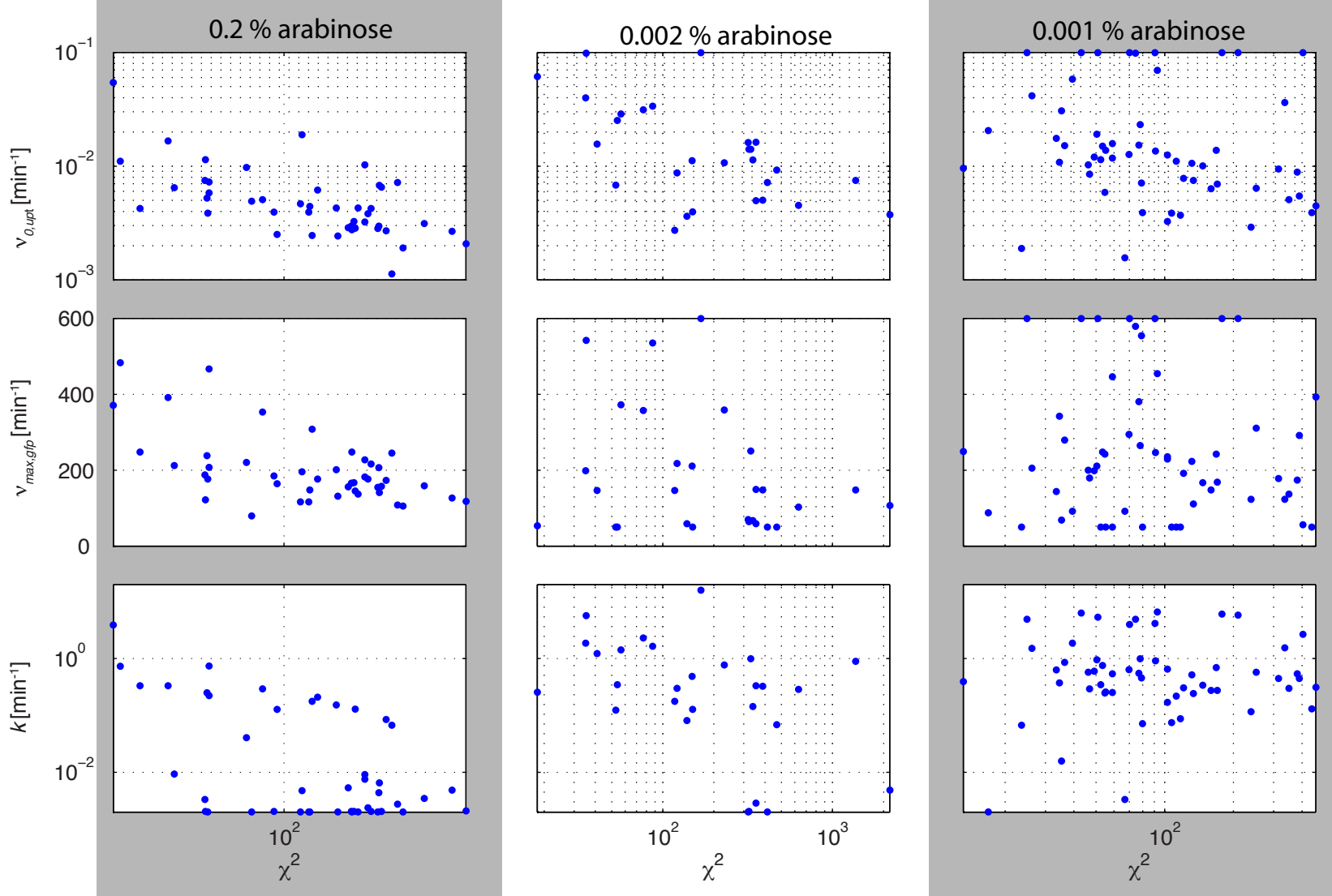

Supplement: Figure S13 — Scatter plots of the fit parameters in Fig. S12 versus the corresponding χ2-value. (PDF) [file pone.0089532.s013.pdf]

0.2 % arabinose

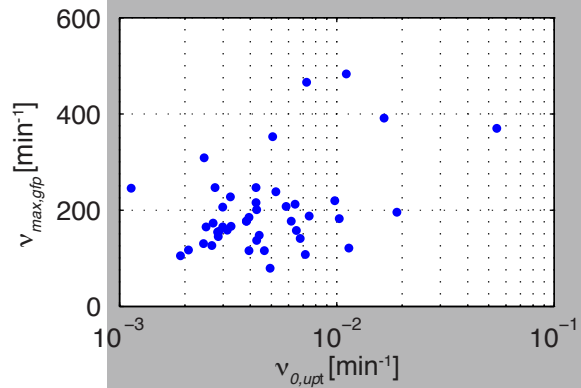

0.002 % arabinose

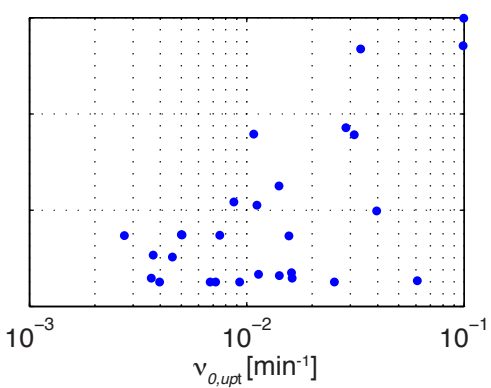

0.001 % arabinose

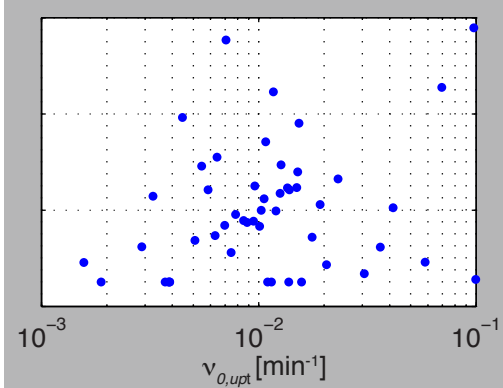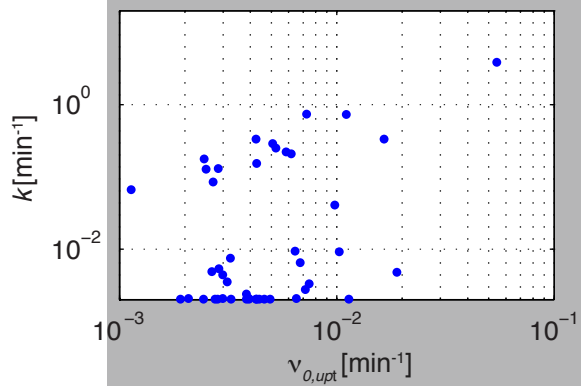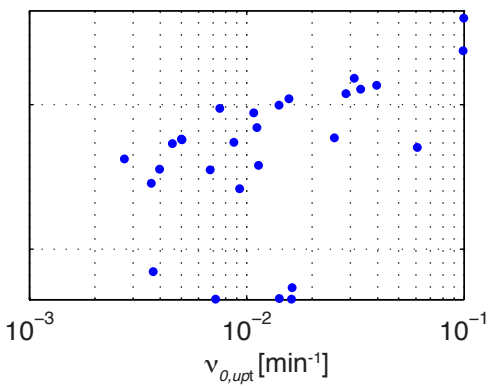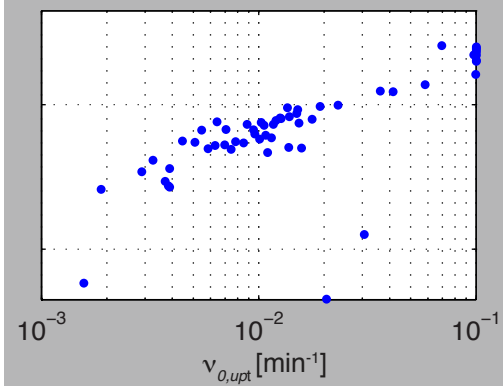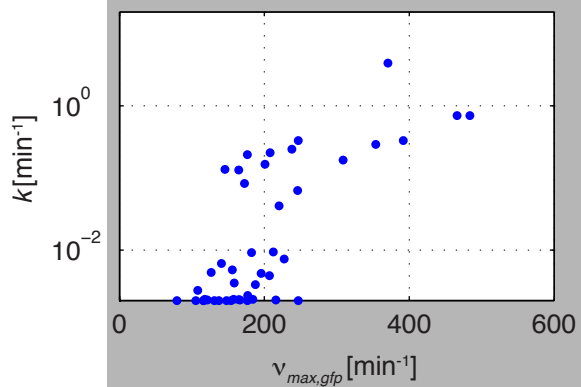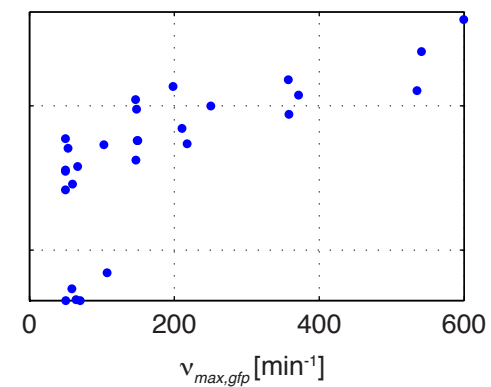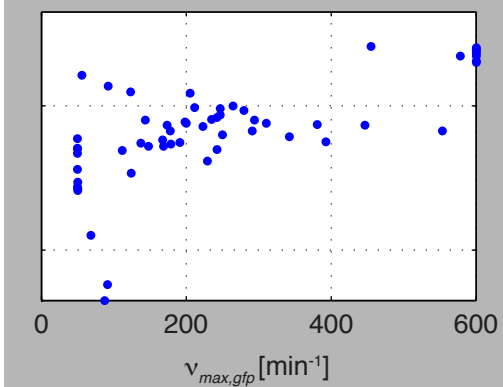

Supplement: Figure S14 — Pairwise scatter plots of individual fit parameters of Fig. S12 against each other. (PDF) [file pone.0089532.s014.pdf]
