# Supplementary material for: Single Cell Kinetics of Phenotypic Switching in the Arabinose Utilization System of E. coli
Source: PLoS One. 2014 Feb 26;9(2):e89532. doi: 10.1371/journal.pone.0089532 (PMC3935871; doi:10.1371/journal.pone.0089532)
Supplement: Table S1 — Bacterial strains used in this study. (DOC) [file pone.0089532.s015.doc]

Table S1. Bacterial strains used in this study.

| Strain and description | Relevant genotype | Source or reference |
| --- | --- | --- |
| MG1655 | F^-^, *λ*^-^, *ilvG*^-^, *rfb*-50, *rph*-1 | (Blattner *et al*., 1997) |
| BW25113 (native *araJ* gene) | F^-^, lacI^q^ rrnBT14 ΔlacZWJ16 hsdR514 ΔaraBADAH33 ΔrhaBADLD78 | (Datsenko and Wanner, 2000) obtained from `E. coli genetic stock center’ (Yale) |
| JW0386-1 (Δ*araJ*) | F^-^, Δ(araD-araB)567, ΔlacZ4787(::rrnB-3), ΔaraJ760::kan, λ^-^, rph-1, Δ(rhaD-rhaB)568, hsdR514 | Keio collection (Baba *et al.*, 2006) obtained from `E. coli genetic stock center’ (Yale) |
| JW1889-3 (native AraE regulation) | F^-^, Δ(araD-araB)567, ΔlacZ4787(::rrnB-3), LAM, ΔaraF751::FRT, rph-1, Δ(rhaD-rhaB)568, hsdR514 rpsL150) | this study |
| JW1889-5 (*araE* under control of P*_lac_*) | F^-^, Δ(araD-araB)567, ΔlacZ4787(::rrnB-3), LAM, ΔaraF751::FRT, P_araE_::P_lac_ rph-1, Δ(rhaD-rhaB)568, hsdR514 rpsL150) | this study |

**REFERENCES**

1. Blattner, F. R., Plunkett, G. 3rd, Bloch, C. A., Perna, N. T., Burland, V., Riley, M., Collado- Vides, J., Glasner, J. D., Rode, C. K., Mayhew, G. F., Gregor, J., Davis, N. W., Kirkpatrick, H. A., Goeden, M. A., Rose, D. J., Mau, B., Y. Shao (1997). The complete genome sequence of *Escherichia coli* K-12. *Science* 277:1453-74.

2. Datsenko, K.A., and Wanner, B, L. (2000). One-step inactivation of chromosomal genes in *Escherichia coli* K-12 using PCR products. *Proc. Natl. Acad. Sci. USA* 97:6640-6645.

3. Baba, T., Ara, T., Hasegawa, M., Takai, Y., Okumura, Y., Baba, M., Datsenko, K. A., Tomita, M., Wanner, B. L., and Mori, H. (2006). Construction of *Escherichia coli* K-12 in-frame, single-gene knockout mutants: the Keio collection. *Mol. Syst. Biol.* 2:0008.
