# Supplementary material for: Single Cell Kinetics of Phenotypic Switching in the Arabinose Utilization System of E. coli
Source: PLoS One. 2014 Feb 26;9(2):e89532. doi: 10.1371/journal.pone.0089532 (PMC3935871; doi:10.1371/journal.pone.0089532)
Supplement: Table S2 — Parameters used in the mathematical model. (DOC) [file pone.0089532.s016.doc]

Table S2. Parameters used in the mathematical model.

| **Parameter** | **Notation** | **Value** | **Source** |
| --- | --- | --- | --- |
| Arabinose uptake velocity | V_max_ | 2000 ara molecules/protein/min | estimated in (Megerle *et al.*, 2008) |
| Michaelis-Menten constant for arabinose uptake via AraE | K_m_ | 0.3 mM | fitted within narrow, physiological range (Daruwalla *et al*., 1981), see Supporting Text S1 for details |
| Arabinose binding threshold | K_a_ | 50 µM | (Schleif, 1969) |
| Maximal transcription rate of P*_E_* | ν_max,e_ | 4.2 min^-1^ | inferred from (Johnson and Schleif, 1995) |
| AraE mRNA degradation rate | λ_e_ | 0.347 min^-1^ | (Johnson and Schleif, 1995) |
| GFP mRNA degradation rate | λ_g_ | 0.116 min^-1^ | (Smolke *et al.*, 2000) |
| AraE translation rate | μ_e_ | 10.4 min^-1^ | unknown; chosen to meet typical burst size of 30 (Thattai and van Oudenaarden, 2001) |
| GFP translation rate | μ_g_ | 3.5 min^-1^ | unknown; chosen to meet typical burst size of 30 (Thattai and van Oudenaarden, 2001) |
| GFP maturation time | τ_m_ | 6.5 min | (Megerle *et al*., 2008) |
| GFP bleaching time | τ_bleach_ | 140 min | measured (this study) |
| Average doubling time | log(2)/γ | 55 min (strain MG1655); 56 min (strain BW25113); 67 min (strain JW0386-1) | measured (this study) |
| Scaling factor between GFP concentration and fluorescence | σ | 0.01 RFU/area/protein | arbitrary |

The basal transporter transcription rates ν_0,e_ (or ν_0,lac_), the GFP expression rate ν_max,gfp_ and the arabinose export rate *k* were estimated from fits to single-cell fluorescence trajectories in Figs. 2 and 5 of the main text. Both their distributions and cross correlations are shown in Figs. S4-S6 and S9-S14. The growth rate γ was determined for individual cells as described in Material and Methods and shown in Fig. S1.

**REFERENCES**

1. Megerle, J. A., Fritz, G., Gerland, U., Jung, K. and Rädler, J. O. (2008). Timing and dynamics of single cell gene expression in the arabinose utilization system. *Biophys. J.* 95:2103-2115.

2. Schleif, R. (1969). Induction of the L-arabinose operon. *J. Mol. Biol.* 46:197-199.

3. Daruwalla, K. R., Paxton, A. T. and Henderson, P. J. (1981). Energization of the transport systems for arabinose and comparison with galactose transport in Escherichia coli. *Biochem. J.* 200, 611-627.

4. Johnson, C. M., and R. F. Schleif. (1995). In vivo induction kinetics of the arabinose promoters in *Escherichia coli*. J. Bacteriol. 177:3438-3442.

5. Smolke, C. D., T. A. Carrier, and J. D. Keasling. (2000). Coordinated, differential expression of two genes through directed mRNA cleavage and stabilization by secondary structures. *Appl. Environ. Microbiol*. 66:5399-5405.

6. Thattai, M., and A. van Oudenaarden. (2001). Intrinsic noise in gene regulatory networks. *Proc. Natl. Acad. Sci. USA.* 98:8614–8619.
