# Supplementary material for: Single Cell Kinetics of Phenotypic Switching in the Arabinose Utilization System of E. coli
Source: PLoS One. 2014 Feb 26;9(2):e89532. doi: 10.1371/journal.pone.0089532 (PMC3935871; doi:10.1371/journal.pone.0089532)
Supplement: Table S3 — Statistics of fitted parameters in Fig. S4. (DOCX) [file pone.0089532.s017.docx]

Table S3. Statistics of fitted parameters in Fig. S4.

|  | Basal transcription rate of genes for uptake proteins ν_0,upt_ [min^-1^] | | | Maximal *gfp* transcription rate ν_max,gfp_ [min^-1^] | | | Arabinose loss rate *k* [min^-1^] | | |  |
| --- | --- | --- | --- | --- | --- | --- | --- | --- | --- | --- |
| Strain (arabinose supply) | 1. Quartile | Median | 3. Quartile | 1. Quartile | Median | 3. Quartile | 1. Quartile | Median | 3. Quartile | |
| BW25113 (0-40 min) | 3,36E-02 | 3,80E-02 | 4,39E-02 | 2,39E+02 | 2,85E+02 | 3,49E+02 | 1,85E+00 | 2,18E+00 | 3,66E+00 | |
| JW0386-1 (0-40 min) | 2,76E-02 | 3,18E-02 | 3,66E-02 | 1,61E+02 | 1,91E+02 | 2,22E+02 | 8,25E-01 | 1,35E+00 | 1,98E+00 | |
| MG1655 (0-40 min) | 2,66E-02 | 3,33E-02 | 3,84E-02 | 8,67E+01 | 1,22E+02 | 1,72E+02 | 1,84E+00 | 2,32E+00 | 3,43E+00 | |
| BW25113 (0-100 min) | 3,00E-02 | 3,22E-02 | 3,81E-02 | 1,88E+02 | 2,13E+02 | 2,88E+02 | 3,27E-03 | 9,37E-02 | 3,00E+00 | |
| JW0386-1 (0-100 min) | 2,87E-02 | 3,33E-02 | 3,60E-02 | 2,90E+02 | 3,48E+02 | 4,02E+02 | 6,46E+00 | 7,15E+00 | 9,05E+00 | |
| MG1655 (0-100 min) | 3,83E-02 | 4,58E-02 | 5,57E-02 | 2,52E+02 | 3,19E+02 | 3,66E+02 | 7,93E+00 | 9,41E+00 | 1,19E+01 | |
