# Supplementary material for: Single Cell Kinetics of Phenotypic Switching in the Arabinose Utilization System of E. coli
Source: PLoS One. 2014 Feb 26;9(2):e89532. doi: 10.1371/journal.pone.0089532 (PMC3935871; doi:10.1371/journal.pone.0089532)
Supplement: Text S1 — Construction of bacterial strains and discussion of modeling rationale. (DOC) [file pone.0089532.s018.doc]

Supplementary Text S1 to:

Single Cell Kinetics of Phenotypic Switching in the Arabinose Utilization System of *E.coli*

Georg Fritz,^† ‡^ Judith A. Megerle,* Sonja A. Westermayer,* Delia Brick,* Ralf Heermann,^‡^ Kirsten Jung,^‡^ Joachim O. Rädler,* and Ulrich Gerland,^†^

Georg Fritz and Judith A. Megerle contributed equally to this work.

^†^Arnold Sommerfeld Center for Theoretical Physics and CeNS, Ludwig- Maximilians-Universität, Munich, Germany; ^‡^Department Biologie I, Bereich Mikrobiologie, Ludwig-Maximilians-Universität, Munich, Germany; *Department for Physics and CeNS, Ludwig-Maximilians-Universität, Munich, Germany

**GENERATION OF *E. COLI* STRAINS**

Cells were grown in LB broth (Sambrock *et al*., 1989) under aerobic conditions at the designated temperature. For solid media, 1.5% (w/w) agar was added. Antibiotics were used at the following final concentrations: ampicillin (50 µg/ml), carbenicillin (50µg/ml in solid media), kanamycin (15µg/ml), and streptomycin (50µg/ml).

The 315 bp intergenic region upstream of *araE* was replaced by the 123 bp intergenic region upstream of lacZ in E. coli strain JW1889-1 using Red/ET-based rpsL genome counter selection (Heermann *et al.*, 2008). As a first step, the kanamycin resistance cassette had to be excised from the JW1889-1 genome. Therefore, E. coli JW1889-1 was transformed with plasmid pCP20 (Cherepanov and Wackernagel, 1995). One colony was picked and incubated in 1 ml LB broth for 3 h at 30°C. Then, temperature was shifted to 37°C and cells were incubated over night. Cells were then plated on LB agar, and single colonies were tested for kanamycin (loss of cassette) and carbenicillin (loss of plasmid pCP20) sensitivity by patching single clones onto LB agar supplemented with kanamycin or carbenicillin, respectively, and the correct clone was named E. coli JW1889-2. Then, the rpsL150 allele was brought into the JW1889-2 genome. E. coli strain JW1889-2 was transformed with plasmid pRed/ET(amp) (GeneBridges, Heidelberg) which encodes encodes the lamda red recombinase system *redαβγ/recT* and was used for Red/ET recombination. rpsL150 allele was amplified by PCR with primers homologous to the rpsL gene (rpsL sense, rpsL antisense) using genomic DNA of E. coli MC4100 (Casabadan, 1976) as template. The linear DNA template was then brought into E. coli JW1889-2 by electroporation using a protocol described elsewhere (Heermann *et al.*, 2008). Single clones were tested for streptomycin resistance and carbenicillin sensitivity [loss of pRED/ET(amp)] by streaking on LB agar supplemented with the respective antibiotics. The correct clone was named E. coli JW1886-3. Then, the intergenic region between araE and kduD containing the araE promoter region was replaced with the rpsL-neo/kan cassette (GeneBridges, Heidelberg) in the JW1886-3 genome. Therefore, the rpsL-neo/kan cassette was amplified by PCR using primers with homology arms that are containing the 50 nucleotides upstream and downstream of the araE/kduD intergenic target region, respectively (araE-rpsL-neo/kan sense, araE-rpsL-neo/kan antisense). E. coli JW1889-3 was transformed with plasmid pRed/ET(amp). The araE-rpsL-neo/kan cassette was then transferred into the E. coli JW1889-3 genome by Red/ET recombination. Single clones were tested for kanamycin resistance, and carbenicillin and streptomycin sensitivity. As wild-type rpsL transferred with the cassette is dominant over the rpsL150 allele, cells that have inserted the rpsL-neo/kan cassette into the genome become streptomycin sensitive again. The correct strain was designated as E. coli JW1889-4. Finally, the rpsL-neo/kan cassette was replaced with the lac promoter region (intergenic region between lacI and lacY) using Red/ET based counter selection. Therefore, the lacI/lacY intergenic region was amplified by PCR using primers with homology arms containing the 50 nucleotides upstream and downstream of the araE/kduD intergenic region (araE-lacP sense and araE-lacP antisense), and then recombined into the E. coli JW1886-4 genome. When the rpsL-neo/kan cassette is replaced with the lacI/lacY intergenic region, due to the loss of the wild-type rpsL gene cells become streptomycin resistant again. Single clones were tested for a kanamycin sensitive, carbenicillin sensitive and streptomycin resistant phenotype. The correct strain was designated as E. coli JW1886-5. The correct recombination events were verified at each step by PCR using primers annealing outside of the respective recombined DNA fragment, and by DNA sequencing.

**DISCUSSION OF MODELING RATIONALE AND PARAMETER VALUES**

For the comparison of our extended model [Eqs. (1)-(7) in the main text] with single-cell fluorescence trajectories, we follow a similar rationale as in ref. (Megerle *et al.*, 2008), where we subsumed all sources of noise during the induction process into the cell-to-cell variability of two key parameters, the delay time τ_d_ and the protein expression rate α_p_. This simplification seemed reasonable, given the significantly lower cell-to-cell variability in all other reaction rates (Megerle *et al*., 2008). Here we adapt this simplification to our extended model of the induction of the ara system and use the basal expression rate of araE, v_0,e_, as a proxy for the (inverse) delay time and choose the maximal transcription rate of P_BAD_, v_max,g_, as a proxy for the protein production rate. The fixation of both parameters to a static value provides a practicable way to capture stochasticity across a population with a simple deterministic model as long as the induction peridod does not exceed the autocorrelation time (~ cell doubling time) in protein levels significantly.

Another key assumption in our analysis is that the arabinose uptake velocity, V_upt_, scales with the external arabinose concentration, a_ex_, according to a Michaelis-Menten process, that is, V_upt_ = V_max_
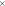
a_ex_/(K_m_+a_ex_), where K_m_ is the Michaelis-Menten constant. Previously, our analysis with the simple gene expression function, in which the transcription rate suddenly switches from `off’ to `on’ once the internal arabinose threshold is reached, pointed to a K_m_ of 2.8 mM. This value is about one order of magnitude larger than literature values (Daruwalla *et al.*, 1981). While there might be biological explanations for such a shift under anaerobic conditions (see Megerle *et al.*, 2008), it is also conceivable that it is a mere artifact of our data analysis with the simplified gene expression function, which did not include arabinose export or metabolism. Therefore, here we vary K_m_ within a physiological range and test which value provides the most coherent explanation for our data. More specifically, we expect that the Michaelis-Menten scaling with external arabinose simultaneously accounts for the modulation in timing (reference strain) and in expression rate (mutant strain), while the distributions of v_0,e_ and v_max,g_ should be independent of the arabinose concentration. Ultimately, the final value of K_m_ = 300 µM found here is well in the range of previous in vivo determinations (Daruwalla *et al.*, 1981).

The value for the maximal arabinose uptake velocity, V_max_, was estimated to be in the range of V_max_ = 200-2000 molecules/protein/min (Megerle *et al*., 2008). While our previous analysis without arabinose efflux required a low number (120 molecules/protein/min) to explain the experimentally observed delay times for reasonable numbers of basally expressed transporters, the presence of the strong efflux in the current model required V_max_ = 2000 molecules/protein/min. This value is also in good agreement with literature values on the lactose/H^+^ symporter LacY (1300-3000 molecules/protein/min) (Wright and Overath, 1984; Dornmair *et al*., 1989).

**REFERENCES**

1. Sambrock, J., Fritsch, E. F. and Maniatis., T. (1989). Molecular Cloning: A Laboratory Manual. Cold Spring Harbor Laboratory, Cold Spring Harbor, NY.

2. Heermann, R., Zeppenfeld, T., and Jung, K. (2008). Simple generation of site-directed point mutations in the *Escherichia coli* chromosome using Red(R)/ET(R) Recombination. *Microb. Cell Fact.* 7:14.

3. Cherepanov, P.P., and Wackernagel, W. (1995). Gene disruption in *Escherichia coli*: TcR and KmR cassettes with the option of Flp-catalyzed excision of the antibiotic-resistance determinant. *Gene* 158:9-14

4. Casadaban, M. J. (1976). Transposition and fusion of the lac genes to selected promoters in Escherichia coli using bacteriophage lambda and Mu. *J. Mol. Biol.* 104:541-555.

5. Megerle, J. A., Fritz, G., Gerland, U., Jung, K., and Rädler, J. O. (2008). Timing and dynamics of single cell gene expression in the arabinose utiliza,tion system. *Biophys. J.* 95:2103-2115.

6. Daruwalla, K. R., Paxton, A. T., and Henderson, P. J. (1981). Energization of the transport systems for arabinose and comparison with galactose transport in Escherichia coli. *Biochem. J.* 200, 611-627.

7. Wright, J. K., and Overath, P. (1984). Purification of the lactose: H+ carrier of Escherichia coli and characterization of galactoside binding and transport. *Eur. J. Biochem.* 138:497-508.

8. Dornmair, K., Overath, P. and Jä̈hnig, F. (1989). Fast measurement of galactoside transport by lactose permease. *J. Biol. Chem.* 264:342-346.

9. Blattner, F. R., Plunkett, G. 3rd, Bloch, C. A., Perna, N. T., Burland, V., Riley, M., Collado- Vides, J., Glasner, J. D., Rode, C. K., Mayhew, G. F., Gregor, J., Davis, N. W., Kirkpatrick, H. A., Goeden, M. A., Rose, D. J., Mau, B., and Y. Shao (1997). The complete genome sequence of *Escherichia coli* K-12. *Science* 277:1453-74.
